# Deficient butyrate-producing capacity in the gut microbiome is associated with bacterial network disturbances and fatigue symptoms in ME/CFS

**DOI:** 10.1016/j.chom.2023.01.004

**Published:** 2023-02-08

**Authors:** Cheng Guo, Xiaoyu Che, Thomas Briese, Amit Ranjan, Orchid Allicock, Rachel A. Yates, Aaron Cheng, Dana March, Mady Hornig, Anthony L. Komaroff, Susan Levine, Lucinda Bateman, Suzanne D. Vernon, Nancy G. Klimas, Jose G. Montoya, Daniel L. Peterson, W. Ian Lipkin, Brent L. Williams

**Affiliations:** 1 Center for Infection and Immunity, Mailman School of Public Health, Columbia University, New York, NY 10032, USA; 2 Department of Biostatistics, Mailman School of Public Health, Columbia University, New York, NY 10032, USA; 3 Department of Epidemiology, Mailman School of Public Health, Columbia University, New York, NY 10032, USA; 4 Division of General Medicine, Department of Medicine, Brigham and Women’s Hospital, Harvard Medical School, Boston, MA 02115, USA; 5 Levine Clinic, New York, NY 10021, USA; 6 Bateman Horne Center, Salt Lake City, UT 84102, USA; 7 Institute for Neuro-Immune Medicine, College of Osteopathic Medicine, Nova Southeastern University, Fort Lauderdale, FL 33314, USA; 8 Miami VA Medical Center, Miami, FL 33125, USA; 9 Palo Alto Medical Foundation, Jack S. Remington Laboratory for Specialty Diagnostics of Toxoplasmosis, Palo Alto, CA 94301, USA; 10 Sierra Internal Medicine at Incline Village, Incline Village, NV 89451, USA; 11 Department of Pathology and Cell Biology, College of Physicians and Surgeons, Columbia University, New York, NY 10032, USA; 12 Lead contact

## Abstract

Myalgic encephalomyelitis/chronic fatigue syndrome (ME/CFS) is characterized by unexplained debilitating fatigue, cognitive dysfunction, gastrointestinal disturbances, and orthostatic intolerance. Here, we report a multi-omic analysis of a geographically diverse cohort of 106 cases and 91 healthy controls that revealed differences in gut microbiome diversity, abundances, functional pathways, and interactions. *Faecalibacterium prausnitzii* and *Eubacterium rectale*, which are both recognized as abundant, health-promoting butyrate producers in the human gut, were reduced in ME/CFS. Functional metagenomics, qPCR, and metabolomics of fecal short-chain fatty acids confirmed a deficient microbial capacity for butyrate synthesis. Microbiome-based machine learning classifier models were robust to geographic variation and generalizable in a validation cohort. The abundance of *Faecalibacterium prausnitzii* was inversely associated with fatigue severity. These findings demonstrate the functional nature of gut dysbiosis and the underlying microbial network disturbance in ME/CFS, providing possible targets for disease classification and therapeutic trials.

## In brief

The metagenomic analysis of Guo et al. reveals extensive gut microbiome dysbiosis in patients with myalgic encephalomyelitis/chronic fatigue syndrome and uncovers differences in species abundance, function, and ecological interactions. Reduced abundance of *Faecalibacterium prausnitzii* is associated with butyrate deficiency and symptom severity, providing insights into the functional consequences of dysbiosis.

## Graphical abstract

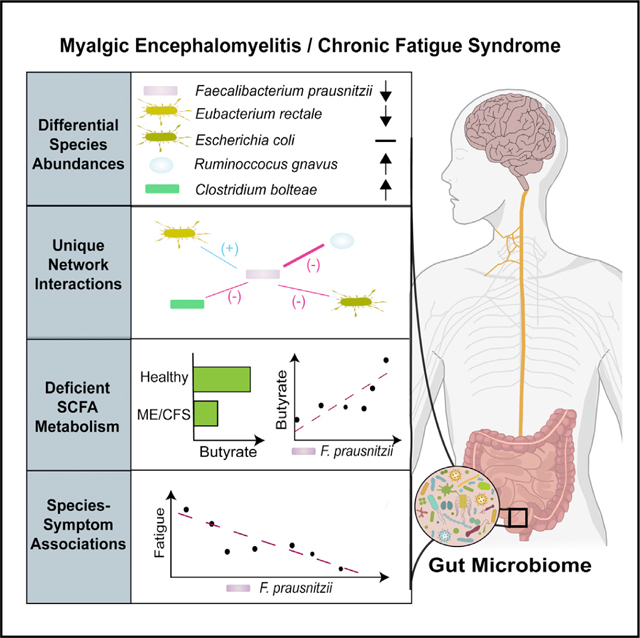

## INTRODUCTION

Myalgic encephalomyelitis/chronic fatigue syndrome (ME/CFS) is an unexplained debilitating and chronic disease characterized by a spectrum of symptoms including fatigue, post-exertional malaise, impaired memory, pain, gastrointestinal dysfunction, immune abnormalities, and sleep disturbances.^1–3^ The global prevalence of ME/CFS ranges between 0.4% and 2.5%. The illness predominantly begins in adults 20–40 years of age and is more common in females than males with a ratio averaging about 3:1, ranging as high as 6:1.^4–7^ In the US alone, this syndrome afflicts 2.5 million individuals.^3^

A diagnosis of ME/CFS is based on clinical criteria and symptoms utilizing different but overlapping case definitions including the 1994 US Centers for Disease Control and Prevention (CDC), Canadian Consensus, International Consensus, and the Oxford and Institute of Medicine criteria.^1–3,8,9^ The cause or causes of ME/CFS are unknown. However, many underlying biological abnormalities have been identified in people with ME/CFS, including defective energy metabolism and a hypometabolic state, redox imbalance, dysregulated immune responses, multiple abnormalities of the central and autonomic nervous system, and multiple autoantibodies, many against targets in the central and autonomic nervous system, as summarized in two recent reviews.^10,11^

Many, but not all, people with ME/CFS report that their symptoms began following an acute prodromal viral-like syndrome.^9^ Post-infection fatigue states are known to develop following viral, bacterial, and protozoal infections.^10^ Clusters of ME/CFS have also followed outbreaks of infectious disease.^12,13^ Interestingly, recent viral outbreaks including Severe acute respiratory syndrome-associated coronavirus (SARS-CoV), Ebola, Middle East respiratory syndrome coronavirus (MERS-CoV), and SARS-CoV-2 have been associated with long-term sequelae that include persistent fatigue and cognitive symptoms consistent with those reported in ME/CFS.^14–18^

The gut microbiome can influence human health and affect physiology through its influence on pathogen resistance, gut barrier maintenance, metabolism, immunity, and neural signaling. The microbiome directly affects immunity via the production of various immunomodulatory metabolites such as short-chain fatty acids (SCFAs), which support immunological tolerance and maintain inflammatory equilibrium.^19–22^ Several studies, including our own, have identified differences in the gut microbiome (dysbiosis) between ME/CFS patients and healthy individuals.^23–29^ However, most of these studies have limitations due to small sample size, insufficient assessment of potentially confounding variables, and biases associated with 16S rRNA gene sequencing.

In this study, we employed shotgun metagenomics and metabolomics to assess dysbiosis in the largest prospective case control study to date, consisting of 106 ME/CFS subjects and 91 matched healthy controls. Our findings provide insights into disturbances in the microbiome and their relationship with fatigue symptoms in ME/CFS.

## RESULTS

### Study population

The study included stool samples from 106 ME/CFS cases who met both the 1994 CDC and the 2003 Canadian Consensus criteria for ME/CFS, and 91 healthy control individuals recruited from 5 sites in the United States (CA, NY, NV, UT, and FL). The site of collection, age, sex, BMI, and demographic index were matched in healthy controls. The characteristics of the ME/CFS patients and healthy participants of this study are outlined in Table 1. More ME/CFS females enrolled in the study (70.8%), consistent with a higher prevalence of ME/CFS in females in the general population. Sex distribution, mean age, and BMI were similar between ME/CFS and healthy controls. No cases or controls took an antibiotic in the 6 weeks prior to stool sample collection (see participant exclusion criteria), and antibiotic use in the 6–12-week period prior to sample collection was similar between cases and controls. More ME/CFS subjects reported frequent prebiotic, probiotic, anti-depressant, and narcotics for pain usage compared with healthy controls.

The majority of the ME/CFS cases were categorized as long duration, presenting with ME/CFS for longer than 3 years. Self-reported irritable bowel syndrome (sr-IBS) diagnosis was more frequent in ME/CFS cases (33.0%) compared with healthy controls (3.3%), consistent with prior reports of higher IBS comorbidity in ME/CFS.^30–32^

### Characterization of the gut microbiome

**Figure 1.**
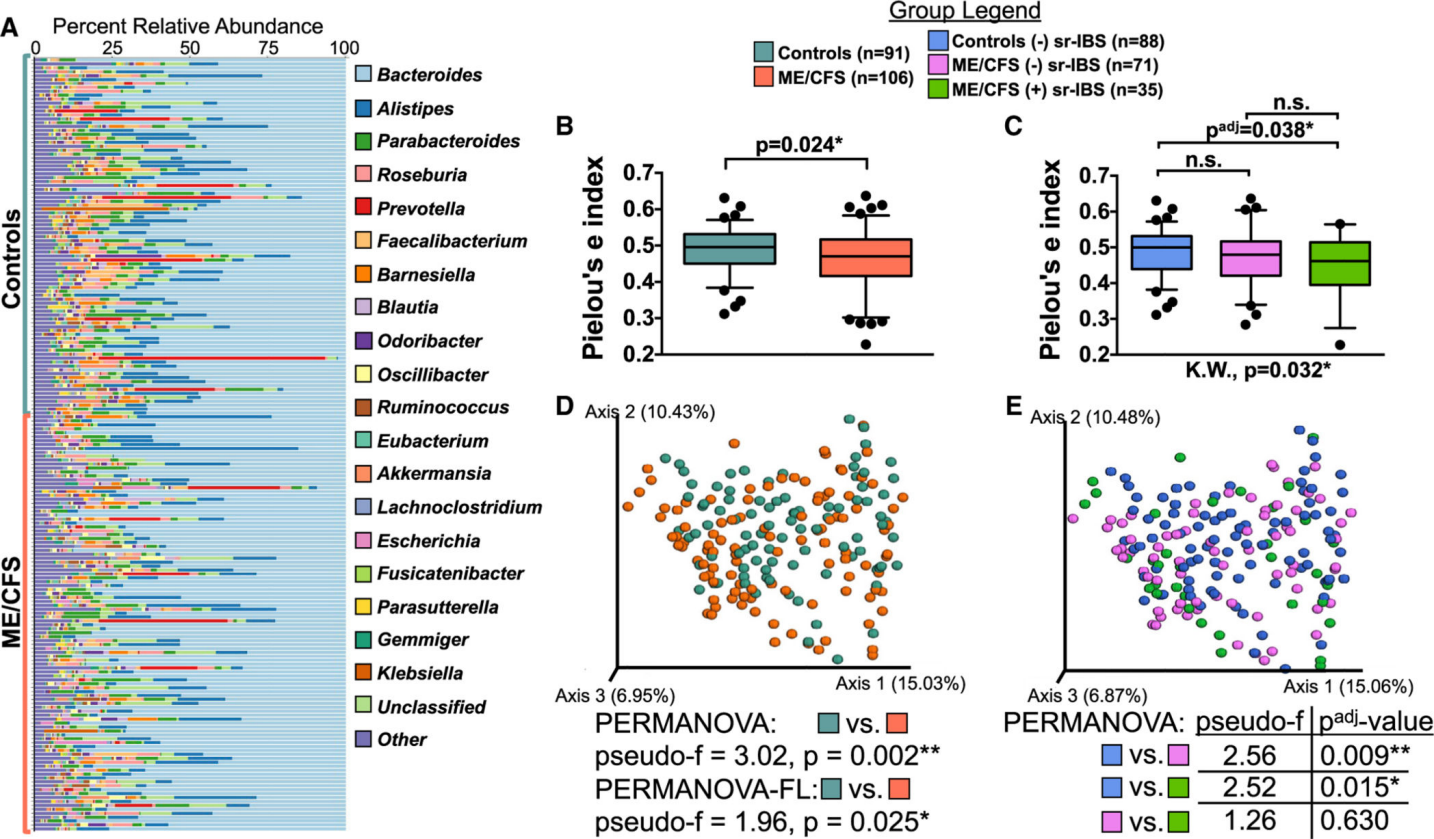
Gut microbiome diversity (A) Genus-level distribution of bacteria in the fecal microbiota of ME/CFS cases (n = 106) and healthy control subjects (n = 91). (B and C) Distribution of microbiota alpha diversity (Pielou’s e index) comparing ME/CFS and healthy controls (B) and groups stratified by sr-IBS status (C). Box-and-whiskers plots represent the interquartile ranges (25th through 75th percentiles, boxes), medians (50th percentiles, bars within the boxes), the 5th and 95th percentiles (whiskers below and above the boxes), and outliers beyond the whiskers (closed circles). See also Figures S1A–S1D. (D and E) PCoA plots of microbiota beta diversity (Bray-Curtis dissimilarity metric) comparing ME/CFS and healthy controls (D) and groups stratified by sr-IBS status (E). Statistical significance: Mann-Whitney U test (B); Kruskal-Wallis test (K.W.), and Mann-Whitney U test with Bonferroni correction (p^adj^ value, C); PERMANOVA and PERMANOVA-FL (D); PERMANOVA with Bonferroni correction (p^adj^ value, E). *p or p^adj^ < 0.05; **p or p^adj^ < 0.01.

Shotgun metagenomic sequencing of fecal samples from all 106 ME/CFS and 91 healthy controls was undertaken. Across the dataset, *Bacteroides* was the most abundant genus in both ME/CFS cases and healthy controls, consistent with prior reports in US populations (Figure 1A).^33^ Other high abundance genera observed in ME/CFS and healthy controls included *Alistipes*, *Parabacteroides*, *Roseburia*, *Prevotella*, and *Faecalibacterium*.

Bacterial alpha diversity metrics (Pielou’s evenness, Shannon index, and observed species-calculated from rarefied data) were compared in univariate analyses between ME/CFS cases and healthy controls. The microbiota of ME/CFS patients had lower evenness and Shannon diversity (Figures 1B and S1A) but similar observed species (Figure S1B) compared to healthy controls.

IBS has been associated with changes in the gut microbiota, including alpha diversity.^34–37^ As the frequency of sr-IBS diagnosis was higher in ME/CFS in this study, and our prior research indicated that comorbid sr-IBS may be associated with alterations in microbiota in ME/CFS,^24^ we performed stratified analyses to assess differences in alpha diversity among healthy controls without sr-IBS (n = 88), ME/CFS without sr-IBS (n = 71), and ME/CFS with sr-IBS (n = 35). As only three healthy controls had sr-IBS diagnosis in our cohort, they were not compared. Evenness and Shannon diversities were only lower in ME/CFS with sr-IBS compared with healthy controls without sr-IBS (Figures 1C and S1C). No differences were found among stratified groups for observed species (Figure S1D). These findings were validated by regression analyses, adjusting for covariates (Tables 2 and S1A). These results indicate that alpha diversity differences are associated with sr-IBS rather than ME/CFS, highlighting the importance of considering IBS as a potential confounder of microbiota differences in ME/CFS.

Differences in microbiota beta diversity between ME/CFS and healthy controls, based on the Bray-Curtis dissimilarity (calculated from rarefied data) were significant, even with adjustment for covariates (Figure 1D). Unlike alpha diversity, stratification by sr-IBS diagnosis revealed that beta diversity differed between healthy controls without sr-IBS and both ME/CFS without sr-IBS and ME/CFS with sr-IBS (Figure 1E). Beta diversity did not differ between ME/CFS without and with sr-IBS. Thus, beta diversity differences between ME/CFS and healthy controls are independent of sr-IBS diagnosis.

As, our findings suggest that some differences in microbiota may be confounded by sr-IBS diagnosis, while others are independent of sr-IBS, we employed both unstratified and stratified analyses and include sr-IBS diagnosis as a covariate, where appropriate, in all subsequent regression models.

### Differential abundance of bacterial species in ME/CFS

**Figure 2.**
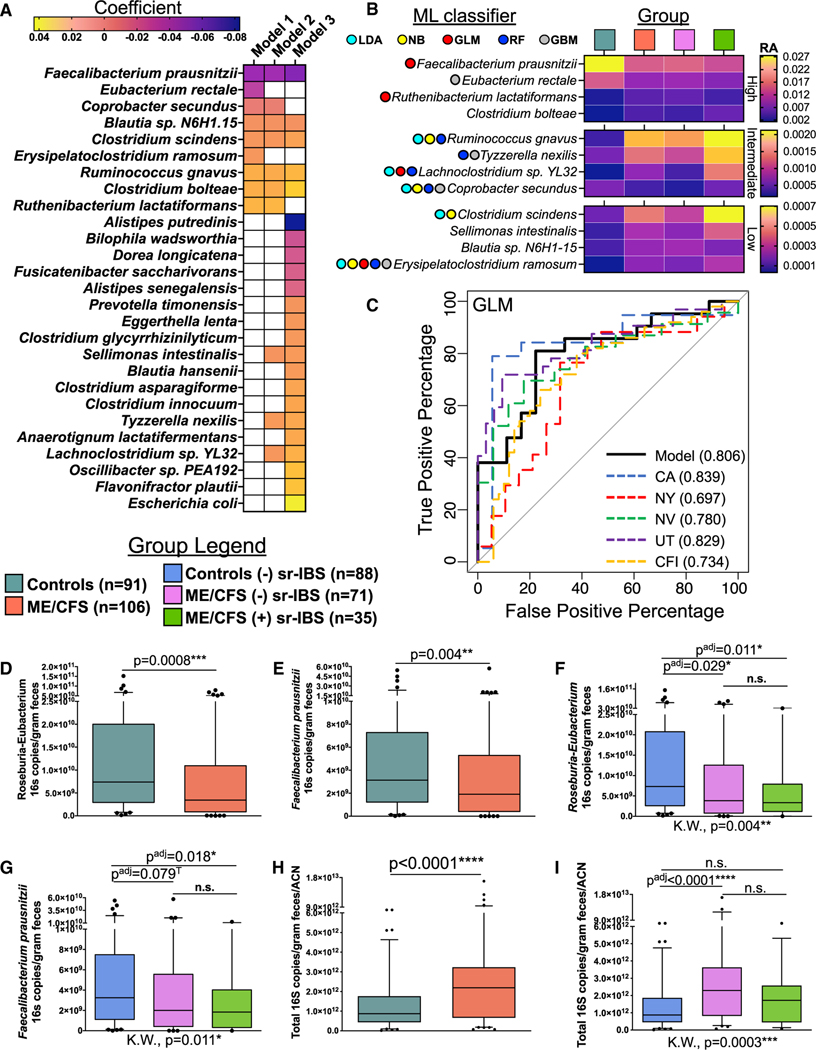
Differential abundance, machine-learning classifiers, and quantitation of fecal bacteria (A and B) Differential abundance. (A) Heatmap showing differentially abundant species identified from models 1–3 (MaAsLin2 analyses). Strength and direction of association are indicated by the color scale of the regression coefficient. FDR adjusted p value (q value) < 0.05 was considered significant; white indicates non-significant associations. See also Table S2. (B) Heatmap showing the average relative abundance (RA) of the twelve ME/CFS-specific species in unstratified and stratified groups, divided into high, intermediate, and low RA. Species selected in each ML classifier model are indicated by colored circles. (C) ML classifiers. Generalized linear model (GLM) receiver operating characteristic (ROC) curves for classification of ME/CFS based on four bacterial species (*F. prausnitzii*, *R. lactatiformans*, *Lachnoclostridium sp*. *YL32*, and *E. ramosum*). AUC values are shown for the primary dataset in this study (model), by geographic sites (CA, NY, NV, and UT), and for the external validation dataset (CFI). See also Figure S2. (D–I) Bacterial quantitation (qPCR). Distribution of *Roseburia-Eubacterium* (D and F) and *F. prausnitzii* (E and G) and total bacterial (H and I) 16S rRNA genes per gram of feces (also normalized for ACN in (H) and (I) between ME/CFS and healthy controls (D, E, and H) and among stratified groups (F, G, and I). Box-and-whiskers plots represent the interquartile ranges (25th through 75th percentiles, boxes), medians (50th percentiles, bars within the boxes), the 5th and 95th percentiles (whiskers below and above the boxes), and outliers beyond the whiskers (closed circles). Statistical significance: Mann-Whitney U test (D, E, and H); Kruskal-Wallis test (K.W.) and Mann-Whitney U test with Bonferroni correction (p^adj^ value, F, G, and I). *p or p^adj^ < 0.05; **p or p^adj^ < 0.01; ***p or p^adj^ < 0.001; ****p or p^adj^ < 0.0001; T, trend (p or p^adj^ < 0.1).

To identify species that differ in relative abundance (RA) in the microbiome of ME/CFS as well as those that distinguish ME/CFS with sr-IBS, we employed Microbiome Multivariable Associations with Linear Models (MaAsLin2)^38^ and compared four models. Model 1 compares all ME/CFS vs. healthy controls. Model 2 compares ME/CFS without sr-IBS vs. healthy controls without sr-IBS. Model 3 compares ME/CFS with sr-IBS vs. healthy controls without sr-IBS. Model 4 compares ME/CFS with sr-IBS vs. ME/CFS without sr-IBS. All models are adjusted for covariates. Model 4 did not reveal significant species differences after false discovery rate (FDR) adjustment (Figure 2A; Table S2).

Model 1 identified 9 differentially abundant species after FDR adjustment. Model 2 identified 10 differentially abundant species (7 overlapping with model 1). Model 3 identified twenty-three differentially abundant species (5 overlapping with model 1; 3 overlapping with model 2) (Figure 2A). All species identified in model 1 and those species identified in both model 2 and model 3 are considered to be ME/CFS specific (independent of sr-IBS and other covariates), while species only identified in model 3 are considered to be specific to the ME/CFS with sr-IBS subgroup. Among ME/CFS-specific species, three species (*F. prausnitzii*, *E. rectale*, and *C. secundus*) were deficient in ME/CFS, and nine species (*R. lactatiformans*, *C. bolteae*, *R. gnavus*, *E. ramosum*, *C. scindens*, *Blautia sp. N6H1.15*, *S. intestinalis*, *T. nexilis*, and *Lachnoclostridium sp*. *YL32*) were enriched in ME/CFS compared with controls.

Among ME/CFS with sr-IBS-specific species (uniquely identified in model 3), five were deficient (*A. putredenis*, *B. wadsworthia*, *D. longicatena*, *F. saccharivorans*, and *A. senegalensis*), and ten were increased (including *E. coli*, *F. plautii*, and *Oscillibacter sp. PEA192*). However, we note that 9 out of the 15 species uniquely identified in model 3 were significantly associated with ME/CFS in model 1 or model 2 before FDR adjustment, including *E. coli* (Table S2). Thus, the specificity of these species to sr-IBS requires further testing.

### Machine learning classifiers trained with differentially abundant species

As MaAsLin2 analysis identified twelve, ME/CFS-specific, differentially abundant species, applying covariate adjustment and rigorous FDR correction, we considered the performance of these twelve species in disease classification using five state-of-the-art machine learning (ML) classifiers; generalized linear model (GLM), random forest (RF), gradient-boosting method (GBM), naive Bayes (NB), and linear discriminant analysis (LDA). In the selected models, combinations of four to five species (4 species for GLM, GBM, and NB; 5 species for RF and LDA) from the twelve species tested performed the best with each ML classifier, indicating improved performance with reduced dimensionality (Figures 2B, 2C, and S2). As an accurate ML classifier should be robust to geographic variation, we evaluated the performance of ML classifiers across geographic sites. Finally, we tested the generalizability of the best performing models using an external validation metagenomic dataset from ME/CFS cases (n = 50) and healthy controls (n = 50) recruited and sampled years earlier from our Chronic Fatigue Initiative (CFI) study.^24^

While all selected models showed good classification performance on the overall primary dataset (GLM: AUC = 0.806, GBM: AUC = 0.804, RF: AUC = 0.799, LDA: AUC = 0.796, and NB: AUC = 0.788, Figures 2C and S2A–S2D, respectively), classifier-dependent variation in performance was observed when evaluating geography-based classification and generalizability to the external CFI dataset. Overall, the GLM and GBM models performed the best when considering both geography (GLM: CA-AUC = 0.839, NY-AUC = 0.697, NV-AUC = 0.780, UT-AUC = 0.829; GBM: CA-AUC = 0.807, NY-AUC = 0.768, NV-AUC = 0.788, UT-AUC = 0.834) and generalizability to the external validation dataset (GLM: CFI-AUC = 0.734; GBM: CFI-AUC = 0.687). The GLM performed slightly better for generalizability, and GBM performed slightly better for geography (Figures 2C and S2A). The improved generalizability in the GLM classifier may be attributable to the combination of species that performed best in the primary dataset. In fact, assessment of differential abundance of the twelve bacterial species in the external CFI dataset showed that ten species differed significantly between controls and cases (Figure S2E). Only *C. secundus* and *T. nexilis* did not differ between cases and controls in the CFI study, neither of which were selected by the GLM classifier, while either one or both of these species were selected by each of the other classifiers.

### Quantitation of BPB in fecal samples

In order to assess quantitative differences in fecal butyrate-producing bacteria (BPB), we carried out qPCR analysis using assays targeting the 16S rRNA genes of *Roseburia*-*Eubacterium* genera and the species *F. prausnitzii*. Compared with healthy controls, ME/CFS subjects had significantly lower 16S rRNA gene copies of *Roseburia*-*Eubacterium* genera (Figure 2D) and the species *F. prausnitzii* (Figure 2E) per gram of feces. In stratified analyses, *Roseburia*-*Eubacterium* 16S copies/g of feces were lower in both ME/CFS subjects without sr-IBS and ME/CFS subjects with sr-IBS compared with healthy controls without sr-IBS (Figure 2F). Similarly, lower quantities of *F. prausnitzii* 16S copies/g of feces were found when comparing ME/CFS without or with sr-IBS to healthy controls without sr-IBS, though only a trend was observed in the former after multiple testing correction (Figure 2G).

As total bacterial load in stool has never been evaluated in ME/CFS, we carried out qPCR using a broad-range 16S rRNA gene-targeting assay. As copy number of the 16S rRNA gene differs among different species, we further adjusted our quantitation by the average 16S rRNA gene average copy number (ACN) per sample.^39^ In contrast to the observed deficiencies in BPB in ME/CFS, ME/CFS subjects had higher quantities of total 16S copies/g of feces/ACN compared with healthy controls (Figure 2H). Stratified analyses revealed that ME/CFS subjects without sr-IBS had higher quantities of total bacterial 16S copies/g of feces/ACN compared with healthy controls without sr-IBS, while other group comparisons did not differ after adjusting for multiple comparisons (Figure 2I).

All qPCR findings were validated by generalized linear regression models, adjusting for covariates (Tables 2 and S1B). The findings indicate that, despite having higher quantities of total bacterial load in feces, ME/CFS patients had significantly lower quantities of important BPB.

### Fecal bacterial functional metagenomic pathways differ between ME/CFS and healthy controls

**Figure 3.**
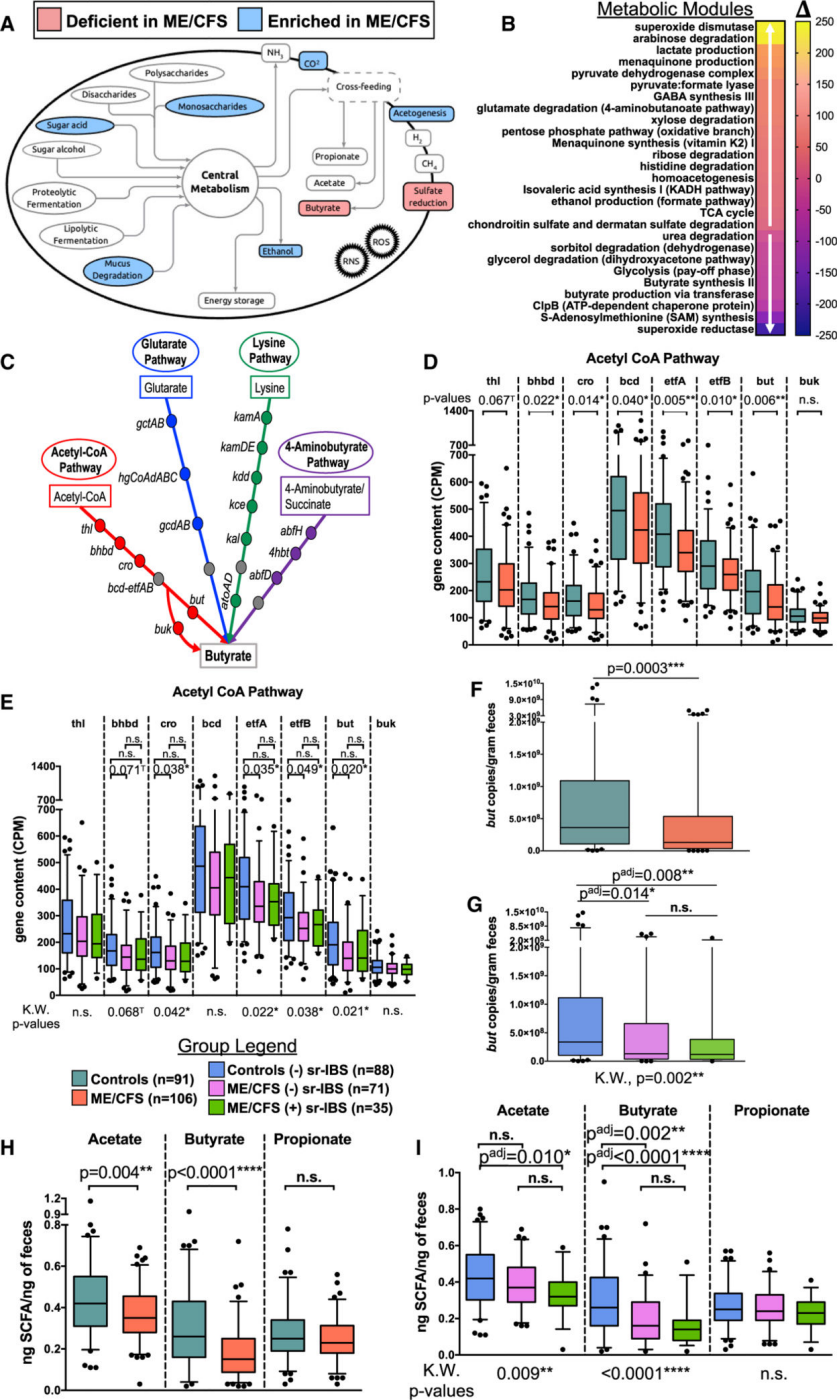
Functional metagenomic and metabolomic analyses (A and B) GoMixer metabolic processes (A) and modules (B) that are deficient (red in A, downward facing white arrow in B) or enriched (blue in A, upward facing white arrow in B) in ME/CFS compared with healthy controls. (B) Color scale indicates the mean difference in modules (Δ) between ME/CFS and controls. An FDR adjusted p value (q value) < 0.1 was considered significant. See also Table S3. (C) Schematic showing the 4 bacterial gene pathways of butyrate production. Note: the bcd-etfAB complex is shared among all 4 pathways (gray circles). (D and E) Distribution of gene counts (CPM) for genes in the acetyl-CoA pathway between ME/CFS and healthy controls (D) and among stratified groups (E). See also Figure S3 and Tables S1C–S1F. (F and G) Distribution of fecal *but* gene copies per gram of feces (qPCR) between ME/CFS and healthy controls (F) and among stratified groups (G). See also Table S1B. (H and I) Distribution of fecal SCFAs (ng SCFA/ng feces) measured by GC-MS between ME/CFS and healthy controls (H) and among stratified groups (I). See also Table S4. Box-and-whiskers plots (D-I) represent the interquartile ranges (25th through 75th percentiles, boxes), medians (50th percentiles, bars within the boxes), the 5th and 95th percentiles (whiskers below and above the boxes), and outliers beyond the whiskers (closed circles). Statistical significance: Mann-Whitney U test (D, F, and H); Kruskal-Wallis test (K.W.) followed by Mann-Whitney U test with Bonferroni correction (p^adj^ value, E, G, and I), where K.W. was significant (p < 0.05). n.s., not significant; *p or p^adj^ < 0.05; **p or p^adj^ < 0.01; ***p or p^adj^ < 0.001; ****p or p^adj^ < 0.0001; T, trend (p or p^adj^ < 0.1).

Functional metagenomic analysis was carried out using GOmixer.^40^ Comparison between ME/CFS subjects and healthy controls revealed nine global metabolic processes that differed between the gut microbiome of ME/CFS subjects and controls (Figure 3A; Table S3A). Two processes, butyrate and sulfate metabolism, were deficient in ME/CFS. Seven processes, CO_2_ metabolism, monosaccharide degradation, acetogenesis, ethanol production, cross-feeding intermediate metabolism, sugar acid degradation, and mucus degradation, were enriched in ME/CFS. At the level of gut metabolic modules, six modules were deficient, and fifteen modules were enriched (Figure 3B; Table S3B). The specific module for *butyrate production* via *transferase* was deficient in ME/CFS, consistent with deficiencies in BPB we observed based on differential abundance and qPCR analyses. Other modules that were deficient in the ME/CFS microbiome included *superoxide reductase* and *sorbitol degradation*. Modules that were enriched in ME/CFS included *superoxide dismutase*, *lactate production*, and *pyruvate dehydrogenase complex*. Analysis of GOmixer gut-brain modules also revealed deficient metagenomic content for *butyrate synthesis II*, along with *ClpB (ATP-dependent chaperone protein)*, and *S-Adenosylmethionine (SAM) synthesis* (Figure 3B; Table S3C). Enriched gut-brain modules in ME/CFS included *Menaquinone synthesis (vitamin K2) I*, *GABA synthesis III*, and *Isovaleric acid synthesis I (KADH pathway)*.

To further evaluate the specific pathways for butyrate production affected in ME/CFS, we investigated the metagenomic gene content for genes along the four pathways of bacterial butyrate production: the acetyl-CoA pathway, the glutarate pathway, the lysine pathway, and the 4-aminobutyrate pathway (Figure 3C). Content of genes along the acetyl-CoA pathway showed deficiencies for nearly every gene in ME/CFS relative to healthy controls (Figure 3D). Quantitatively, the majority of bacteria in the intestine encode and utilize *but* rather than *buk* as the terminal gene in the acetyl-CoA pathway, including *Eubacterium*, *Roseburia*, and *Faecalibacterium* genera.^41^ Few genes in the other pathways were deficient in ME/CFS compared with healthy controls (Figures S3A–S3C). Even in stratified analyses by sr-IBS status, most genes along the acetyl-CoA pathway remained significantly depleted in the microbiome of ME/CFS subjects without sr-IBS compared with healthy controls without sr-IBS (Figure 3E). No genes in the other pathways differed significantly among groups in stratified analyses (Figures S3D–S3F). These findings were validated with generalized linear regression, adjusting for covariates (Tables 2 and S1C–S1F).

We also quantitated the fecal *but* gene, the dominant terminal gene in the bacterial acetyl-CoA pathway, by qPCR.^42^ The *but* copies/g feces were lower in ME/CFS compared with control subjects (Figure 3F), independent of sr-IBS status (Figure 3G). This finding was further validated using generalized linear regression models, adjusting for covariates (Tables 2 and S1B).

### Assessment of SCFAs in feces

Given the strong evidence indicating reduced abundance and quantity of BPB and reduced metagenomic capacity for producing butyrate, we measured fecal SCFAs. In univariate analyses, the concentration of both acetate and butyrate were lower in ME/CFS compared with healthy controls, while propionate was unchanged (Figure 3H). In stratified analyses, acetate was lower in ME/CFS subjects with sr-IBS compared with healthy controls without sr-IBS but did not differ in ME/CFS subjects without sr-IBS. In contrast, butyrate was lower in both ME/CFS subjects without sr-IBS and with sr-IBS compared with healthy controls without sr-IBS (Figure 3I). These findings were validated with generalized linear regression models, adjusting for covariates. Unlike our prior regressions, the ME/CFS*sr-IBS interaction term was significant (Table S4A). Thus, we evaluated stratified regression models, adjusted for covariates (bottom 3 rows in Tables S4A–S4D). The results indicate that both deficient acetate and butyrate are associated with ME/CFS with or without sr-IBS, while deficient propionate is only found in ME/CFS subjects with sr-IBS. There is a larger effect size in terms of the estimated fold change for fecal butyrate concentrations compared with either acetate or propionate.

### Differential associations of bacterial species with fecal SCFA levels in ME/CFS

**Figure 4.**
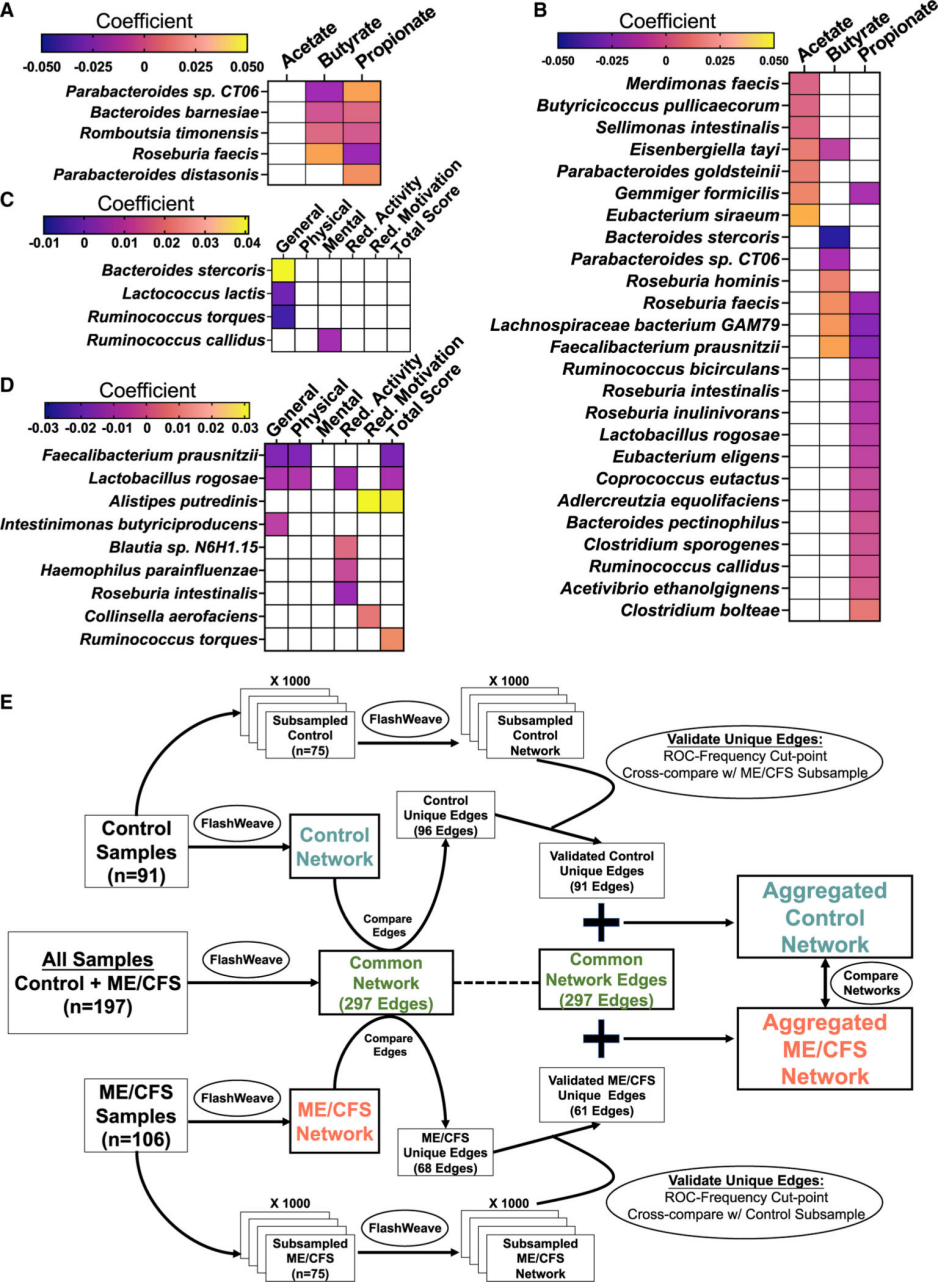
Association of species abundances with SCFAs and fatigue symptoms and co-abundance network analysis strategy (A–D) Heatmaps showing associations between the relative abundance of species and molar proportions of individual SCFAs (A and B) and between species and individual dimension and total fatigue scores (MFI: C and D) among healthy controls (A and C: n = 91) and among ME/CFS cases (B and D: n = 106) (MaAsLin2 analyses). Strength and direction of the associations are indicated by the color scale of the regression coefficient. FDR was controlled with a p value cutoff < 0.01; white indicates non-significant associations. See also Tables S5 and S6. (E) Schematic showing the staged strategy for bacterial, species-level co-abundance network analysis employed for this study. See also STAR Methods.

Given the differences we found in butyrate pathways, and the deficient levels of SCFAs in ME/CFS, we sought to determine whether bacterial associations with SCFA levels differ in ME/CFS. To these ends we employed MaAsLin2 GLMs with adjustment for covariates, including sr-IBS. In healthy controls (Figure 4A; Table S5A), no individual species were associated with fecal acetate. The largest effect sizes indicated that *R. faecis* was positively associated with butyrate and negatively associated with propionate. *P. distasonis* and *Parabacteroides sp*. *CT06* were positively associated with propionate, and *P. sp*. *CT06* was negatively associated with butyrate.

In ME/CFS subjects (Figure 4B; Table S5B), MaAsLin2 revealed substantially more associations. Of note, *E. siraeum* and *G. formicilis* had the largest positive coefficient associated with fecal acetate; *F. prausnitzii* had the largest positive and *B. stercoris* had the largest negative coefficient associated with butyrate; *R. hominis*, *R. faecis*, and *Lachnospiraceae bacterium GAM79* were also positively associated with butyrate. Many bacterial species were negatively associated with propionate, including *F. prausnitzii*, *Lachnospiraceae bacterium GAM79*, *R. faecis*, and *G. formicilis*, which were all positively associated with either butyrate or acetate. Only *C. bolteae* was positively associated with propionate.

These results indicate that in ME/CFS, more individual bacterial species have direct positive or negative associations with fecal SCFAs. These include *F. prausnitzii*, which had the largest positive effect on butyrate in ME/CFS subjects, was deficient in ME/CFS compared with controls and is one of the most abundant BPB in the human gut.

### Relationships among bacterial species abundance and fatigue scores

In order to determine whether bacterial species abundance has any relation to symptom severity, we evaluated the relationship among bacterial species and fatigue scores based on the five dimensions of the multidimensional fatigue inventory (MFI), using MaAsLin2 with covariate adjustment. A higher score indicates more severe fatigue in each dimension. Severe fatigue is a symptom unique to ME/CFS in this study, and median scores in all five dimensions differed substantially between cases and controls (Table 1).

In healthy controls, *B. stercoris* and *R. torques* were positively associated, while *L. lactis* was negatively associated with general fatigue, and *R. callidus* was positively associated with mental fatigue (Figure 4C; Table S6).

Among ME/CFS subjects, *F. prausnitzii* and *L. rogosae* were negatively associated with multiple fatigue dimension scores (Figure 4D). The greatest effect was observed for *F. prausnitzii*, having the largest negative coefficients. *A. putredinis* was positively associated with reduced motivation and total MFI scores. *Blautia sp. N6H1.15* (also a species increased in ME/CFS: Figures 2A and 2B) was positively associated with and *L. rogosae*, *R. intestinalis*, and *H. parainfluenzae* were negatively associated with reduced activity. *C. aerofaciens* was positively associated with reduced motivation scores. *R. torques* was positively associated with total MFI scores.

Overall, these findings indicate that fatigue symptoms and severity in ME/CFS are associated with distinct bacterial species, especially *F. prausnitzii*.

### Healthy controls and ME/CFS cases harbor distinctive species co-abundance networks

While diversity metrics and differential abundance can capture broad differences in the composition of the microbiome, the gut microbiome is an ecological community characterized by complex interactions among resident species. To investigate group-specific differences in inter-species relationships, we employed a staged strategy to infer co-abundance networks using Flashweave^43^ that adjusts for covariates, maximizes sample size from our cohort by building intermediate networks (the common network, the control network, and the ME/CFS network), includes subsampling steps to validate “unique” species interactions and exclude spurious associations in each group, and derives two final aggregated networks for comparison (the aggregated control network [AggCN] and the aggregated ME/CFS network [AggMN]) (details of the strategy, intermediate networks, and unique edges can be found in Figures 4E, S4, and S5 and STAR Methods; additional metrics describing all networks, unique edges, and complete edge lists for all networks are available in Table S7).

**Figure 5.**
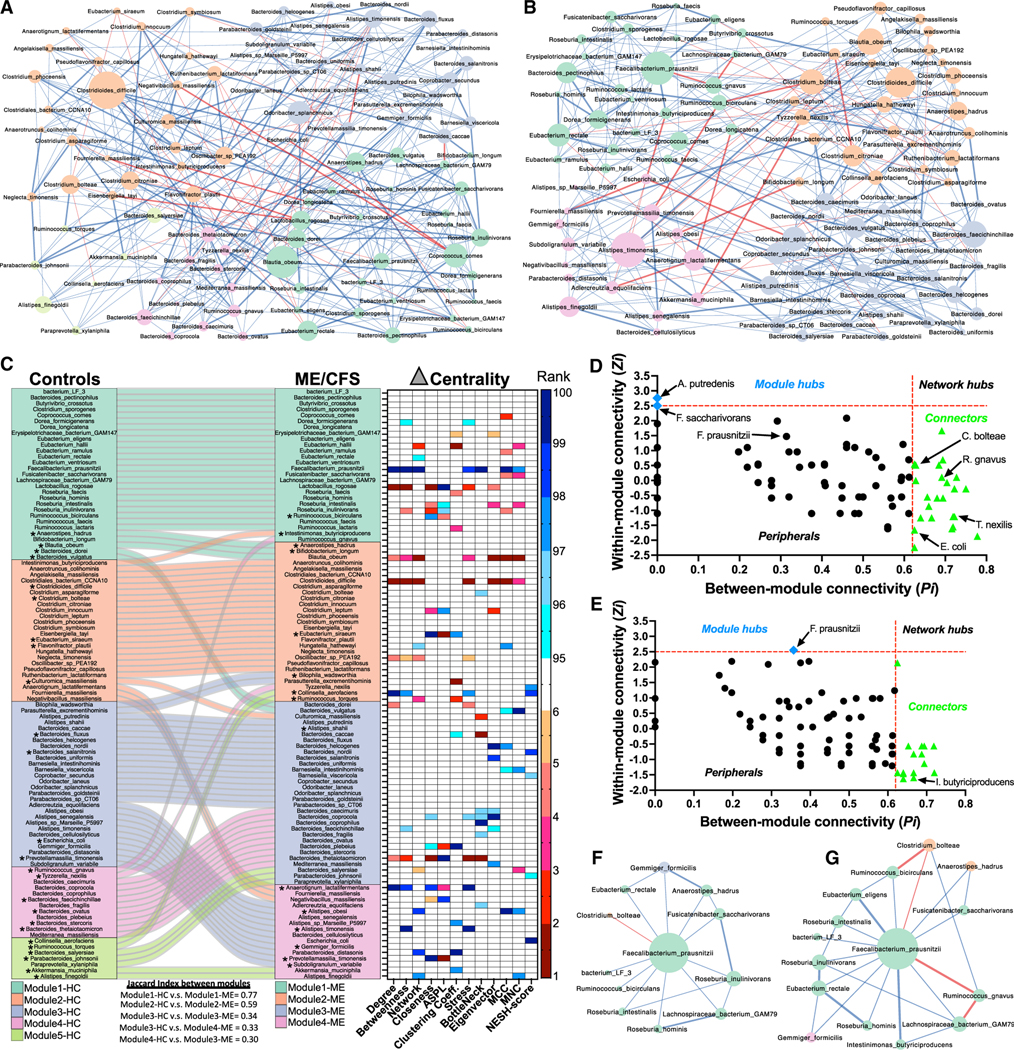
Species co-abundance network comparisons between groups (A and B) Species co-abundance networks derived from the AggCN (A) and AggMN (B). Nodes are sized by betweenness centrality and colored according to their community module membership. Blue edges indicate positive and red edges negative correlations; thicker edges highlight unique edges within each group. (C) Alluvial plot showing the shuffling of species between community modules identified in the AggCN (controls, left) and the AggMN (ME/CFS, right). Jaccard index between group modules is shown below the plot. Asterisks indicate connector species identified in (D) and (E). The associated heatmap shows top ranked species based on difference in centrality metrics (delta centrality). Only top ranked changes are shown (≥95^th^ percentile = higher centrality in ME/CFS [blue scale] or ≤5^th^ percentile = higher centrality in controls [red scale]). ASPL, average shortest path length; MCC, maximal clique centrality; MNC, maximum neighborhood component; NESH, neighbor shift score. (D and E) Zi-Pi plot showing within-module and between-module connectivity for each of the 100 species (each point represents a species) in the AggCN (D) and the AggMN (E). Cutoff zones defining species as network hubs, module hubs, connectors, and peripherals are indicated (dotted red lines and zone labels). Blue diamonds, module hubs; green triangles, connector species; black circles, peripherals. (F and G) Subgraphs derived from the first-order neighbors of *F. prausnitzii* and their edges in the AggCN (F) and AggMN (G). Nodes are sized by their degree and their colors reflect their module membership. Blue edges indicate positive and red negative correlations; thicker edges highlight unique interactions within each group.

The final derived AggCN (Figure 5A) and the AggMN (Figure 5B) were compared. All 100 species (nodes) were present in both networks, with 388 edges in the AggCN and 358 edges in the AggMN (additional metrics are in Table S7). Community clusters or modules were identified in both AggCN and AggMN (Figure 5C). Five community modules were identified in the AggCN, while only four communities were identified in the AggMN. A high degree of shuffling of species between community modules was identified in ME/CFS compared with healthy controls, arising from the unique set of interactions we identified in both groups. In total, 42 species shuffled into different ME/CFS modules. As one example, in module1, which contains the majority of butyrate producers, including *F. prausnitzii*, and shares the highest similarity (Jaccard index = 0.77) between healthy controls and ME/CFS, two new members shuffled into module1-ME from module2-HC (*I. butyriciproducens*) and from module4-HC (*R. gnavus*). These membership changes are likely attributable to increased connectivity (unique interactions) of these two species to module1 members in the ME/CFS network, including *F. prausnitzii* (shown in Figure S4E).

Local node (species) topology differences were evaluated between the aggregated networks to characterize the nodes with the largest differences in centrality measures (delta centrality, Figure 5C, heatmap). *F. prausntizii* had the highest ranked degree, betweenness, network, bottleneck, and maximal clique centrality (MCC) and among the highest stress and maximum neighbor centrality differentials (higher centrality scores in the AggMN compared with the AggCN). Other species with high ranked centrality differentials included *C. aerofaciens*, *A. lactatifermentans*, and several other species belonging to module4-ME. Species with higher centrality metric scores in the AggCN included *L. rogosae* in module1-HC, *B. obeum*, *C. difficile* in module2-HC, and *B. thetaiotamicron* in module4-HC. The highest neighbor shift score (NESH), indicating potential “drivers” of network differences, was found for *E. coli*, followed by *C. aerofaciens*, *B. nordii*, *T. nexilis*, and *B. viscericola*.^44^

The within-module connectivity (*Zi*) and the between-module-connectivity (*Pi*) of species revealed that only *A. putredenis* from module3-HC and *F. saccharivorans* from module1-HC were identified as module hubs in the AggCN (Figure 5D). In the AggMN, only *F. prausnitzii* was identified as a module hub (Figure 5E), indicating that *F. prausnitzii* has increased connectivity to other species within module1-ME. Many of the species that were identified as connectors in either group (asterisks in Figure 5C: i.e., *I. butyriciproducens*, *R. gnavus*, *E. coli*, and *T. nexilis*) shuffled between modules. This suggests that many connectors, with low *Zi* and *high Pi*, are more prone to module shuffling, driven by establishment of unique between-module interactions or the loss of unique within-module interactions.

To better understand how co-abundance network disturbances in ME/CFS may influence *F. prausnitzii*, we evaluated the network neighborhood around *F. prausnitzii* in the AggCN and the AggMN. The subgraphs representing the first-order *F. prausnitzii* neighborhood (nodes directly connected to *F. prausnitzii*) and their corresponding edges from the AggCN (Figure 5F) and the AggMN (Figure 5G) revealed more nodes and edges and more negative edges in the AggMN. Two intermodular connections were present in both networks, a positive edge to *G. formicilis*, an acetate producer, and a negative edge to *C. bolteae* (the only negative interaction in the AggCN). While all unique edges in the AggCN (3 edges) were positive and indirect (not directly connected to *F. prausnitzii*), there were more unique edges in the AggMN (7 edges), forming more direct edges with *F. prausnitzii*, including connection to two species, *R. gnavus* (which forms a new direct negative edge with *F. prausnitzii*) and *I. butyriproducens*, that shuffled into module1-ME in the AggMN network. Both *R. gnavus* and *C. boteae* also introduce two indirect negative edges in the AggMN. Although directionality of edges cannot be inferred in these networks, these interactions are intriguing as both *C. bolteae* and *R. gnavus* had increased RA, while *F. prausnitzii* was decreased in ME/CFS (Figures 2A and 2B). Although the proportion of positive and negative edges in the total AggCN and AggMN did not differ (Fisher’s exact test, p = 0.427, Figures S6A–S6C), in the second-order neighborhood of *F. prausnitzii*, the proportion of negative edges to positive edges in the AggMN was significantly higher than for the AggCN (Fisher’s exact test, p value = 0.033). Additionally, the proportion of unique negative to unique positive edges in the second-order neighborhood was also significantly higher in the AggMN compared with the AggCN (Fisher’s exact test, p value = 0.006). These results indicate that, in ME/CFS, more negative species interactions are in the first- and second-order network neighborhood of *F. prausnitzii*.

## DISCUSSION

### Species abundance differences and their application as disease classifiers

The genera *Faecalibacterium*, *Eubacterium*, and *Roseburia* are prominent BPB in the human GI tract, with *F. prausnitzii* and *E. rectale* being two of the most abundant and prevalent species.^41^ Both the RAs of these species and the absolute fecal load (qPCR) of BPB were deficient in fecal samples from ME/CFS patients. The only other species identified with reduced RA in ME/CFS was *C. secundus*, an acetate producer, that could contribute to the net acetate deficiency we also found in ME/CFS subjects.^45^ An additional nine species had increased RA in ME/CFS compared with healthy controls. The most abundant among these nine species are *R. lactatiformans*, *C. bolteae*, and *R. gnavus*. *R. lactatiformans* is a lactate producer.^46^ Although we did not measure fecal lactate, functional metagenomic analysis identified the metabolic module for lactate production as being increased in ME/CFS relative to healthy controls. Increased fecal *C. bolteae* (synonym: *Enterocloster bolteae*) abundance has been associated with autism and some autoimmune and atopic diseases.^47–51^ In multiple sclerosis, the abundance of fecal *C. bolteae* correlates positively with fatigue.^50^ In this study, *C. bolteae* was positively associated with fecal propionate only in ME/CFS subjects, while many butyrate producers were inversely associated with propionate. *R. gnavus* is a mucin-degrading bacteria, which has been associated with inflammatory bowel disease, produces secondary bile acids, which can affect overall community composition in the gut, and produces an inflammatory polysaccharide.^52,53^ We found an enrichment in the metabolic process for mucus degradation in ME/CFS from functional metagenomic analyses. *R. gnavus* also formed an inverse network edge with *F. prausnitzii* in the ME/CFS but not the control network.

In addition to their functional importance, these twelve species could serve as biomarkers for disease classification, as their associations with ME/CFS in this cohort are independent of a range of potentially confounding covariates. Our study is unique for its sampling of cases and controls across different geographic sites in the US, and we find that our ML models are robust to geographic variation and generalizable to an external validation cohort (CFI study).

An additional, unexpected finding from our qPCR analyses was that ME/CFS patients have higher total bacterial load in feces than healthy controls. Antibiotics, small intestinal bacterial overgrowth associated with IBS, or specific diets (i.e., fermentable oligosaccharides, disaccharides, monosaccharides and polyols diets) could influence bacterial load.^54–57^ However, we controlled for antibiotics and sr-IBS in our study. While diets could differ between ME/CFS subjects and controls, we note that more ME/CFS subjects than controls reported taking prebiotic fiber supplements, which typically stimulate the growth and activity of BPB and were controlled for in our study. A plausible explanation for this observation may be derived from the depletion of butyrate and its antimicrobial and anti-inflammatory properties. Butyrate stimulates expression of endogenous antimicrobial peptides and antimicrobial activity in intestinal macrophages.^58,59^ The depletion of intestinal butyrate and loss of these regulatory functions in ME/CFS could diminish control over bacterial growth.

### Fecal SCFA deficiencies and their associations with bacterial species

Butyrate is an important health-promoting bacterial metabolite with diverse beneficial properties, including its role in host energy metabolism. Butyrate serves as the primary energy source for colonocytes, accounting for 70% of the energy obtained by epithelial cells in the colon.^60^ In addition, butyrate influences the proliferation of intestinal epithelial and stem/progenitor cells, suppresses cancer cell proliferation, and can influence epigenetic changes as a histone deacetylase inhibitor. Butyrate also plays a role in promoting epithelial barrier function by regulating Hypoxia-Inducible Factor (HIF)-1 and tight junction proteins.^61^ Finally, butyrate also mediates important immunomodulatory functions in the intestine by promoting regulatory T cells, inhibiting inflammatory cytokine production, and inducing antimicrobial activity in macrophages.^59^ Thus, deficiency in this intestinal homeostatic metabolite could contribute to a range of detrimental physiological disturbances including a weakened epithelial barrier and enhanced intestinal inflammation. Evidence for disruption of intestinal barrier function is supported by prior research showing elevated levels of plasma lipopolysaccharides in ME/CFS, indicative of microbial translocation.^27^

In addition to butyrate, ME/CFS subjects have reduced quantities of fecal acetate. Metabolic cross-feeding between bacteria is an important ecological interaction in bacterial communities that influences network structure and composition. Acetate produced by bacterial fermentation of carbohydrates or acetogens is utilized by BPB for butyrate production, and BPB may grow poorly in the absence of acetate.^62–64^ Thus, net acetate deficiency may contribute to deficient BPBs and butyrate and may be associated with known acetate producers such as *C. secundus*, which was lower in RA in ME/CFS; or *E. siraeum* and *G. formicilis*, which were positively associated with fecal acetate in ME/CFS. *G. formicilis* also shares a positive edge with *F. prausnitzii* in our networks. In contrast to butyrate and acetate, propionate was only reduced in patients with sr-IBS. Prior research has shown decreased concentration of fecal propionate in constipation-predominant IBS.^65,66^

### Relationships among bacterial species and fatigue symptoms

In contrast to healthy controls, species-symptom associations among ME/CFS cases are more extensive. *F. prausnitzii* and *L. rogosae* have the most associations with individual dimension scores and total MFI scores. The quantity of fecal *F. prausnitzii* based on qPCR was also inversely correlated with general fatigue, physical fatigue and reduced activity (data not shown). Unfortunately, little is known about *L. rogosae*, which is likely misclassified to the genus *Lactobacillus* (tentative renaming: *Lachnospira rogosae*), precluding discussion of its potential relevance in the microbiome.^67^ *F. prausnitzii* and *L. rogosae* also had the largest network differences in centralities between controls and cases, suggesting that their interactions with other species in the microbiome have changed. *Blautia sp. N6H1.15*, which was positively associated with reduced activity, was also increased in abundance in ME/CFS. *I. butyriciproducens*, which was inversely associated with general fatigue, was also found to have some unique network interactions in ME/CFS, including a direct positive edge with *F. prausnitzii* and a direct negative edge to *E. coli*.

The functional importance of *F. prausnitzii* in the intestine may extend beyond its role as a BPB to include additional anti-inflammatory effects through its production of microbial anti-inflammatory molecule (MAM) as well as salicylic acid. As a potential biosensor of human health, deficiency of fecal *F. prausnitzii* has been associated with a range of other conditions including IBD, IBS, celiac disease, colorectal cancer, obesity, and more recently in patients during and even after recovery from infection with SARS-CoV-2.^68–74^ In a large cohort, the Flemish Gut Flora Project, *Faecalibacterium* along with *Coprococcus* were associated with higher quality of life scores.^75^ Patients with IBD and fatigue have reduced levels of fecal *F. prausnitzii* compared with IBD patients without fatigue, further supporting the association of this bacterium with fatigue symptoms.^76^

It is unclear why *F. prausnitzii* is found in association with so many conditions. It could relate to its physiological sensitivity to environmental perturbations. Both *F. prausnitzii* and *E. rectale* are strict anaerobes, highly sensitive to oxygen and reactive oxygen species which may be produced by other microbes or host inflammation. This may be reflected in our functional meta-genomic findings, showing a reduction of superoxide reductase and an increase in superoxide dismutase in ME/CFS. Additional sensitivities to pH changes, concentrations of bile salts, or nutrients in the gut environment may contribute to reduced fitness.^77^ Another hypothesis may be derived from the high degree of connectivity *F. prausnitzii* has to other species in the microbiome, especially if many of those interactions reflect dependencies. If that were the case, having a high degree of dependence-connectivity may be deleterious, as peripheral disturbances that affect any one of *F. prausnitzii*’s many interacting neighbors could affect its fitness. While not direct evidence, we find an increase in negative edges in the species co-abundance neighborhood of *F. prausnitzii*, as well as a first-order negative interaction with *R. gnavus*. A further explanation for the deficiency of *F. prausnitzii* across many conditions may be commonalities in sickness behavior. The debilitating fatigue experienced by people with ME/CFS reduces physical activity to a level substantially lower than seen in healthy controls.^78–80^ In fact, individuals with ME/CFS may engage in less high-intensity physical activity than even sedentary control subjects.^81^ Several studies, though mostly in animal models, have found that physical activity/exercise has a substantive effect on the composition and function of the gut microbiome including BPB and SCFAs.^82,83^ Thus, differing symptoms across diseases may lead to common behavioral adjustment to those symptoms (i.e., reduced physical activity) resulting in similar changes in the microbiome. Nonetheless, even if deficient butyrate-producing capacity is a consequence rather than a direct cause of symptoms, such a deficiency could both exacerbate ME/CFS-specific symptoms or potentiate the risk for the development of additional health-related issues.

### Ecological network disturbances in ME/CFS

The gut microbiome is a complex ecological community teeming with diverse inter-species interactions that can be beneficial (i.e., mutualism, commensalism, and cross-feeding) or harmful (i.e., parasitism, amensalism, competition for resources or space, and biomolecular warfare) to their fitness. Disturbances in microbiome networks have been reported for other conditions.^84–86^ We find that there is extensive rewiring of the microbiome in ME/CFS, reflected in unique species-species relationships and changes in module membership. Some species become more centralized in the ME/CFS network, establishing more connections (i.e., *F. prausnitzii*), while others establish fewer connections (i.e., *L. rogosae*). We also find that negative interactions between species have increased proximity to *F. prausnitzii* in the ME/CFS network, including a direct negative edge between *F. prausnitzii* and *R. gnavus* and negative interactions affiliated with *C. bolteae* and *E. coli*. While it is tempting to conclude that these negative interactions are driving *F. prausnitzii* deficiency, co-abundance analysis cannot distinguish the direction of interactions, and additional testing would be needed to substantiate such a conjecture.

### Independent corroboration of some key findings

Our manuscript has been co-submitted along with the manuscript of Xiong et al. to *Cell Host & Microbe*.^87^ Each study is distinct, with differences in overall study design and methodology, and each provides unique insights into ME/CFS. However, some key findings and conclusions are corroborated between the two studies. Both groups employ fecal shotgun metagenomics, report reduced abundance of *F. prausnitzii* in ME/CFS, and conclude that there is a reduced genomic capacity for butyrate production in the ME/CFS microbiome. Independent substantiation of findings supports the rigor and reproducibility of each group’s work.

### Limitations of the study

The majority of ME/CFS subjects in this cohort had a long duration of illness (>3 years), and analyses were cross-sectional, precluding evaluation of disturbances occurring early in the disease course or their longitudinal stability in individuals. However, differential abundance of the majority of species we report here were confirmed in an independent external validation cohort. While geographic diversity of sampling is distinctive to this study, both the primary cohort and external validation cohort were derived from individuals from the same five sites in the US. Thus, additional assessment with expanded geographic range is needed to further assess the generalizability of our models. Though we adjust for a range of important covariates in our study, consideration of other potentially confounding factors including dietary factors, medications (in addition to antidepressants and pain narcotics which were considered here), lifestyle factors, and other comorbidities is also warranted.

## Conclusions

Our study comparing the fecal microbiome in ME/CFS subjects and healthy controls matched for age, sex, geography, and socioeconomic status demonstrates significant differences in bacterial diversity, abundances, function, SCFA metabolism, and co-abundance network topology. Species, including the prominent BPB, *F. prausnitzii*, are associated with the severity of fatigue symptoms in ME/CFS subjects. These findings provide unique insights into microbiome disturbances and the functional consequences of dysbiosis that may contribute to the manifestation of symptoms in ME/CFS and identify potentially actionable targets for disease classification and therapeutic testing.

## STAR★METHODS

### RESOURCE AVAILABILITY

#### Lead contact

Further information and requests for resources and reagents may be obtained from the lead contact, Brent L. Williams (bw2101@cumc.columbia.edu).

#### Materials availability

Requests for resources may be obtained from the lead contact, Brent L. Williams (bw2101@cumc.columbia.edu).

#### Data and code availability

Host subtracted, shotgun metagenomic sequences from fecal samples of all ME/CFS and healthy control samples are available from the Sequence Read Archive (SRA) under SRA: PRJNA751448. All additional relevant data are available in this article and its supplemental information files, or from the corresponding author upon request.

Any additional information required to reanalyze the data reported in this paper is available from the lead contact upon request.

### EXPERIMENTAL MODEL AND SUBJECT DETAILS

#### Study Population

The initial cohort consisted of 177 ME/CFS cases and 177 healthy community controls prescreened at five geographically-diverse ME/CFS clinics across the USA (Incline Village, NV; Miami, FL; New York, NY; Palo Alto, CA; Salt Lake City, UT) as part of a National Institutes of Health-sponsored R56 study. ME/CFS cases met the requirements of both the 1994 CDC^1^ and the 2003 Canadian consensus criteria^8^ for ME/CFS. Community control participants were matched to ME/CFS cases based on geographical/clinical site, sex, age, race/ethnicity, and date of sampling (±30 days).

The CDC Criteria require that cases have fatigue persisting for greater than six months that is clinically-evaluated, persistent or relapsing, and which meets five criteria: is of new onset, is not the result of ongoing exertion, is not alleviated by rest, is made worse by exertion, and results in substantial reduction in previous levels of activity. The CDC criteria additionally require the concurrent occurrence of at least four of the following symptoms for at least six consecutive months: sore throat, tender cervical or axillary lymph nodes, muscle pain, multiple joint pain without swelling or redness, headaches of new or different type, unrefreshing sleep, post-exertional malaise, and impaired memory or concentration. The Canadian consensus criteria impose additional restrictions, requiring at least two neurologic/cognitive manifestations, and at least one clinical feature from two of the following three categories: autonomic manifestations, neuroendocrine manifestations, and immune manifestations.

Eligible cases must also have had a diminished or restricted capacity to work, reported a viral-like prodrome prior to onset of ME/CFS, and met a low-score threshold for two out of the following three dimensions measured by the self-reported Short Form-36 General Health Survey (SF-36): vitality <35, social functioning <62.5, role-physical <50. Additional exclusion criteria for both cases and controls included disorders or treatments resulting in immunosuppression, and antibiotic use within six weeks prior to the baseline assessment. Based on these screening criteria, we excluded five ME/CFS cases and one control participant prior to the baseline assessment.

In the current study, a nested sub-cohort was established for participants with complete survey data collection and biospecimen (stool) collection at the first and fourth (final) timepoints for the overall study. Participants were frequency-matched on key demographic elements to ensure similarity between the nested cohort and full cohort. This sub-cohort is comprised of 106 ME/CFS cases and 91 healthy controls that met these criteria; the derivation of this sub-cohort is outlined in Figure S7. All subjects provided written consent in accordance with study protocols approved by the Columbia University Medical Center Institutional Review Board (IRB).

#### Clinical Assessments

All subjects completed standardized screening instruments to assess medical history, family medical history, current medication use, symptom scores, and demographic/lifestyle information, as well as a baseline SF-36. All subjects underwent a screening blood draw to determine that they had normal values in the following three laboratory tests from Quest Diagnostics: complete blood count with differential, comprehensive metabolic panel, thyroid stimulating hormone (TSH).

Subjects eligible for participation after the baseline visit returned to the same clinic four times over the course of one year for sample collection and completion of survey instruments. At each of the four visits subjects completed a range of rating scales, including the Multidimensional Fatigue Inventory (MFI). Prior to all four study visits, subjects were provided with at-home collection kits for stool. Stool samples were collected within 48 hours prior to each study visit and refrigerated until the day of the visit. All samples were shipped to the Columbia University laboratory site in insulated Styrofoam boxes with frozen and refrigerated gel packs. The stool samples were aliquoted, weighed and moved to −80 °C freezers for storage. Only samples and clinical rating scale data obtained at the first study visit were analyzed as part of the present study.

On the medical history questionnaire, participants were asked to self-report if they received an IBS diagnosis (sr-IBS) by a physician and the date of diagnosis. In the analytic sub-cohort, 35 out of the 106 (33.0%) ME/CFS cases reported sr-IBS, while only 3 out of the 91 control subjects (3.3%) reported sr-IBS. The use of probiotic and prebiotic supplements was specifically included in the “current medication use” data collection instrument, which determined the frequency and recent use of these products. Any subjects that indicated consumption of these supplements daily or a few times a week and reported using them within the last week prior to their study visit would be endorsed for that type of supplement. Participants also reported any antibiotic use that occurred outside of the six-week window for study eligibility.

The MFI consists of a 20-item self-reported questionnaire that evaluates five dimensions of fatigue: general, physical and mental fatigue, reduced activity, and reduced motivation.^96^ The scoring for this instrument was transformed into a 0–100 scale to allow for comparisons between dimensions: a score of 100 was equivalent to maximum disability or severity and a score of zero was equivalent to no disability or disturbance. Total MFI scores were calculated by summing the scores from each of the five dimension scores.

### METHOD DETAILS

#### Fecal DNA Extraction

A modified protocol of the QIAmp DNA Stool Mini Kit (Qiagen Inc; Valencia CA, USA) was used for the extraction of DNA from stool samples. Prior to the initiation of the extraction process, all disposable elements including tubes, columns, 0.1mm and 0.5 mm glass beads (MoBio Laboratories) were UV irradiated twice at a distance of 1 inch from UV bulbs and at 3000 × 100 μJ/cm^2^ in a SpectroLinker XL-1500 UV crosslinker (Spectronics Corporation). All liquid extraction reagents in the kit were aliquoted into 2 mL tubes in a UV hood at ≤1 ml and UV irradiated as mentioned above. To confirm that bacterial 16S rDNA contaminants were not introduced from the extraction reagents, two empty 2 mL tubes were used for each set of DNA extraction to serve as negative controls throughout the entire process. Each fecal sample was weighed and < 220 mg of each fecal sample was re-suspended in AL Buffer (Qiagen). Glass beads (0.1mm and 0.5mm, MoBio Laboratories) were added to the mixture. To ensure Gram-positive bacteria lysis, samples were disrupted by bead beating in a TissueLyser (Qiagen) for 5 minutes at 30Hz and incubated for 5 minutes at 95°C. The remaining steps for DNA extraction were performed in a UV hood by following the manufacturer’s protocol. To determine DNA concentration and purity, a NanoDrop ND-100 spectrophotometer (NanoDrop Technnologies, Wilmington, DE) was used and the extracted DNA samples were stored at −80°C.

#### Shotgun metagenomic sequencing and bioinformatic analyses

Shot-gun metagenomics sequencing was carried out on DNA extracts obtained from 197 fecal samples (106 ME/CFS cases and 91 healthy controls). For Illumina library preparation, genomic DNA was sheared to a 200-bp average fragment length using a Covaris E210 focused ultrasonicator. Sheared DNA was purified and used for Illumina library construction using the KAPA Hyper Prep kit (KK8504, Kapa Biosystems). Sequencing libraries were quantified using an Agilent Bioanalyzer 2100. Sequencing was carried out on the Illumina HiSeq 4000 platform (Illumina, San Diego, CA, USA). Sequencing libraries from samples of cases and controls were grouped into eight different sequencing pools. Each sample yielded 27.8 ± 10.3 (mean ± sd) million reads. The demultiplexed raw FastQ files were adapter trimmed using Cutadapt.^88^ Adaptor trimming was followed by the generation of quality reports using FastQC and filtering with PrinSEQ.^89^ Host background levels were determined by mapping the filtered reads against the human genome using Bowtie2 mapper.^90^ After the step of host subtraction, 25.1 ± 9.0 (mean ± sd) million reads per sample remained on average. Non-host reads were subjected to Kraken2 for taxonomy classification.^91^ Kraken2 matches each K-mer within a query sequence to the lowest common ancestor (LCA) of all genomes in the database containing the given K-mer. Our Kraken2 local database included all 16,799 fully sequenced and representative bacteria species genomes in the RefSeq database (December 2018).

The species-level taxonomy abundances were estimated using Bracken, which is recommended to perform a Bayesian estimation of taxonomy abundance after the use of Kraken2.^91,92^ Structural zeros in the abundance table were further identified using the program Analysis of Microbiome Data in the Presence of Excess Zeros version II (ANCOM-II).^93^ The three groups we have compared in our study include ME/CFS with sr-IBS, ME/CFS without sr-IBS, and healthy controls. The taxa presenting as structural zeros in all three categories were eliminated from the dataset. The data was rarefied prior to all alpha and beta diversity analyses using a depth of 500,000 reads. Diversity metrics (alpha diversity: Shannon index, Pielou’s evenness, and observed otus; beta-diversity: Bray-Curtis dissimilarity) were calculated and plotted using the core-diversity plugin and the emperor plugin within QIIME2.^94^ The beta diversity significance among groups was examined by QIIME2 diversity plugin with PERMANOVA tests. To evaluate fecal metabolic functional profiles from SMS data, KEGG gene family abundances and the functional pathway and module abundances were calculated using FMAP with the default database.^95^ The abundance of genes involved in butyrate synthesis was calculated by aligning host subtracted sequence reads to a curated database specifically consisting of butyrate synthesis pathway related genes,^41^ using customized scripts from the FMAP pipeline.

#### qPCR

Plasmid DNA standards were constructed using published qPCR primers targeting the bacterial 16S rRNA gene of *Faecalibacterium prausnitzii* and *Eubacterium*/*Roseburia* genera,^97^ the bacterial *but* gene (BcoA),^42^ and broad-range bacterial 16S rRNA genes to quantitate total bacteria^98^ for absolute quantitation. Bacterial gene targets were first amplified by conventional PCR from human stool samples. PCR products were run on 1% agarose gels and purified using the QIAgen Gel Extraction kit. Purified products were ligated into pGEM T-Easy vector (Promega), transformed into DH5a competent cells, and cultured on Luria Bertani plates with ampicillin. Colonies were inoculated into Luria broth with ampicillin (5 ml), and plasmids were extracted with Pure Link Plasmid Extraction Kit (Invitrogen). After verifying that plasmid sequences had 100% nucleotide similarity to the target bacterial taxa or the bacterial *but* gene, plasmids were linearized with the restriction enzyme SphI, and 10-fold serial dilutions were generated ranging from 5 × 10^6^ to 5 copies (note for *Eubacterium*/*Roseburia* standards, two plasmids were combined consisting of one clone matching *Eubacterium hallii* and one clone matching *Roseburia hominis*). Salmon sperm DNA at 2.5 ng/ml was spiked into the standard dilutions as background DNA. Real time PCR was performed with the ABI StepOne Plus Real Time PCR system (Applied Biosystems). Amplification plots for all plasmid standards were sensitive down to five copies and standard curves generated yielded correlation coefficients ranging from 0.998 to 1. For probe-based assays (total bacteria), each 25 mL real time PCR reaction consisted of 1x TaqMan Universal PCR Master Mix (Applied Biosystems), 300 nM primers, 200 nM probe and 5 ng of fecal DNA. The total bacteria probe was labeled with a 5’-end fluorescent reporter (6-carboxyfluorescine, FAM) and had a 3’-end black hole quencher (BHQ1a, Operon). For SYBR green-based assays, each 25 ml reaction consisted of 1X SYBR Green PCR master mix (Applied biosystems), 300 nM primers and 5ng of fecal DNA. Cycling conditions consisted of 50°C for 2 min, 95° C for 10 min, and 45 cycles at 95°C for 15 s and 60°C for 1 min 30 s (for total bacteria, *Eubacterium*/*Roseburia* 16s *and Faecalibacterium* 16s assays). For *but* gene the cycle conditions were 50°C for 2 min, 95° C for 10 min, and 45 cycles at 95°C for 15 s, 53°C for 1 min 45 s and 77°C for 30 s (data collection). All samples were run in duplicate and averaged for each assay. Absolute quantity was determined based on the weight of fecal samples and expressed as copy numbers per gram of feces. As different species of bacteria encode variable copies of 16S rRNA genes in their genomes, differences in total 16S rRNA gene copies in feces may reflect differences in bacterial load or may reflect differences in the proportions of species with higher or lower 16S rRNA gene copies per bacterial cell. To account for this, we determined the 16S rRNA gene Average Copy Number (ACN) for each individual directly from our unassembled metagenomic sequencing data using the acn.sh tool.^39^ For the total 16S rRNA gene qPCR, we adjusted for 16S rRNA copy number differences by dividing the qPCR quantitated 16S rRNA gene copies per gram of stool by the ACN for each sample.

#### Fecal SCFA metabolomics

Fecal SCFAs were measured by gas chromatography-mass spectrometry (GC-MS) as previously described^99^ with minor modifications. Fecal samples were homogenized and 10 mg resuspended in 0.5 ml 2-ethylbutyric acid (100 μg/ml; as internal standard 1) in LC/MS grade water. The sample was acidified with 0.1 ml concentrated hydrochloric acid, vortexed and placed on ice. 1 ml of 4-methylvaleric acid (100 μg/ml; as internal standard 2) in tert-butyl methyl ether (MTBE) was added, the sample was vortexed and shaken for 30 min at room temperature, and centrifuged for 2 min at 14,000 rcf. Supernatant was collected after centrifugation and 0.1 g of anhydrous sodium sulfate was added to it, vortexed and incubated at room temperature for 10 min. Finally, 100 μl of the supernatant was combined with 25 μl N-tert-Butyldimethylsilyl-N-methyltrifluoroacetamide (MTBSTFA). Calibration standards containing acetic acid, n-butyric acid, and propionic acid as well as the internal standards, 2-ethylbutyric acid and 4-methylvaleric acid, were prepared and derivatized with MTBSTFA. After adding MTBSTFA as a derivatization reagent and incubating for 30 min at 80°C followed by 1 hour at room temperature, samples were injected with a 100:1 split into an Agilent GC7890B gas chromatograph coupled with an Agilent 5977MS mass spectrometer detector. Helium was used as a carrier gas. Analyses were performed using a DB5 duraguard capillary column (30 m × 0.25 mm × 0.25 μm film thickness). The column head pressure was 9.83 psi. Injector, source and quadrupole temperatures were 250°C, 290°C, 150°C, respectively. The GC oven program was 50°C for 0.5 min, increased to 70°C for 3.5 min at 5°C/min, increased to 120°C at 10°C/min, and increased to 290°C for 3 min at 35°C/min. The total run time was 20.857 min.

### QUANTIFICATION AND STATISTICAL ANALYSIS

#### Cohort characteristics

Differences in cohort characteristics between ME/CFS and healthy control subjects were assessed using the Mann-Whitney U test for continuous variables and either the Fisher’s Exact test or Chi-squared test for categorical variables and p < 0.05 was considered statistically significant.

#### Box-and-whiskers plots, Mann–Whitney *U*-test and Kruskal-Wallis test

All box-and-whiskers plots shown in the main and supplementary figures represent the interquartile ranges (25th through 75th percentiles, boxes), medians (50th percentiles, bars within the boxes), the 5th and 95th percentiles (whiskers above and below the boxes), and outliers beyond the whiskers (closed circles). Plots were created and statistical analyses performed using Prism 7 (GraphPad Software, CA). All statistics based on data presented in box-and-whiskers plots comparing all ME/CFS subjects and healthy controls are two-tailed *P*-values derived from Mann–Whitney *U*-tests. For stratified analyses of more than two groups, significance was first determined based on the Kruskal-Wallis test. If the Kruskal-Wallis test was significant at p < 0.05, then between group significance was determined based on the Mann-Whitney U test with multiple testing (Bonferroni) correction applied, and an adjusted p-value (p^adj^) < 0.05 was considered significant.

#### Beta diversity

Beta diversity was assessed based on the Bray-Curtis dissimilarity metric using the rarefied data table. Differences in beta diversity using Principal coordinate analyses (PCoA) were visualized with 3-D plots using QIIME2. Permutational multivariate analysis of variance (PERMANOVA) with 999 Monte Carlo permutations was used to evaluate the statistical significance of the dissimilarity between groups. The Freedman-Lane PERMANOVA test^100^ was used to assess differences in microbiota beta diversity between ME/CFS and healthy controls, and was adjusted for covariates (sr-IBS status, site of sampling, sex, BMI, race/ethnicity, age, antibiotic usage 6–12 weeks prior to sample collection, probiotic supplement use, and prebiotic supplement use). Two-group comparisons were considered significant at p < 0.05. For stratified analyses (more than two groups), the FDR was controlled by Bonferroni correction and an adjusted p-value (p^adj^) < 0.05 was considered significant.

#### Microbiome co-abundance network analysis

Species-level co-abundance networks were inferred using Flashweave.^43^ Flashweave employs a local-to-global learning approach to predict direct interactions in networks from heterogeneous microbial datasets. In addition to its inference of direct interactions through conditional independence tests, Flashweave has several benefits over other methods including the use of compositionality correction and use of partial correlation and mutual information, allowing for covariate adjustment to remove associations that may be confounded by metavariables. All networks were constructed in Flashweave-S mode with default settings, including FDR adjustment; only edges with p^adj^ < 0.05 are included in networks. We used Flashweave’s built-in adapted centered log ratio (clr-adapt) method to transform our unrarefied read counts table. Only species with minimum percent relative abundance ≥ 0.02% and prevalence in ≥ 40% across the dataset were included in the analyses (100 species after filtration) to reduce the sparsity of the dataset and spurious correlations. This filtering selected only the 100 most prevalent and abundant species in both groups: controls (minimum prevalence of any species = 81.3%, maximum prevalence = 100%, average prevalence +/− SD = 99.3% +/− 2.9%) and ME/CFS (minimum prevalence of any species = 68.9%, maximum prevalence = 100%, average prevalence = 98.7% +/− 4.6%). Nearly all species in controls (97/100) and ME/CFS cases (96/100) had > 90% prevalence in each group. For all constructed Flashweave networks we adjust the partial correlation with the following covariates to remove associations that may be confounded by these variables: sr-IBS diagnosis, site of recruitment, sex, BMI, race and ethnicity, age, antibiotic use within 6–12 weeks of testing, probiotic supplement use, prebiotic supplement use, and sample sequencing depth (continuous covariates were z-transformed). Deriving co-abundance networks from sample sets of different size can limit inferences that can be drawn from their comparison, as smaller sample sizes tend to produce smaller networks and more spurious associations. In this study, we use a staged strategy to maximize our sample size for inferring both shared and unique interactions in the co-abundance networks of controls and ME/CFS subjects, and employ subsampling with equal sample size for validating their differences (schematic shown in Figure 4E). First, we use Flashweave to derive a “Common Network” by including all samples (n=197; 91 controls + 106 ME/CFS cases). In addition to the above-mentioned covariates, we also include disease status as a covariate only for this Network. The Common Network inferred from this analysis consisted of all 100 species (nodes) included in the analysis and contained 297 edges (diameter = 5, radius =4, density = 0.06) (Figure S4A; Table S7). Our intent in deriving this network is to maximize our sample size to infer the most robust bacterial associations that are shared between healthy controls and ME/CFS and independent of all other included covariates.

Next, we use Flashweave to derive the stratified, group-specific networks using only the controls (n=91, “Control Network”) and only the ME/CFS cases (n=106, “ME/CFS Network”), including all covariates except disease status. Edges from these two networks are cross-compared with the “Common Network” to identify the “Unique” edges in each group (those not present in the “Common Network”). Although our sample sizes differ between groups, we maximize sample size in this stage to maximize edge retrieval from each group. The individual Control Network (Figure S4B; Table S7: 99 nodes, 210 edges, diameter = 7, radius = 5, density = 0.04) and ME/CFS Network (Figure S4C; Table S7: 100 nodes, 223 edges, diameter = 6, radius = 4, density = 0.05) produced smaller networks with lower density compared to the Common Network, consistent with the effects of smaller sample sizes on correlation-based network inference. Compared with the Common Network, the Control Network had a lower Edge Jaccard Index (EJI = 0.29) than the ME/CFS Network (EJI = 0.42) (Table S7), owing to the greater number of candidate “Unique” edges in the Control Network (96 candidate “Unique” Control edges) compared to the ME/CFS Network (68 candidate “Unique” ME/CFS edges).

The robustness of these “Unique” edges are then validated by subsampling each group without replacement at equal sample size (n=75) with 1000 iterations for each group. Networks were inferred from each of these 2000 subsamples with Flashweave, including all covariates. Edge retrieval frequency in the Control and Case subsampled networks were determined for all edges found in the “Common Network”, all “Unique” edges found in the Control Network and Case Network, as well as the frequencies of edges not found in any of the three networks (“edges not found in Networks”). From the total subsampled Control Networks and ME/CFS Networks, 1499 and 2155 distinct edges were obtained, respectively, with frequencies of edge retrieval ranging from 1/1000 to 1000/1000 subsampled networks in each group. The distributions of frequencies between groups varied modestly (Kolmorov-Smirnov test, p = 0.031), largely attributable to a higher proportion of low frequency edges in the ME/CFS subsample (Figure S5A). Within both the Control and ME/CFS subsampled networks, edges present in the Common Network (median retrieval frequency: 95% CI, Control subsample-257: 152–329, ME/CFS subsample-348: 282–394) and the candidate “Unique” edges from each group (Control subsample-536: 457–648, ME/CFS subsample-342: 272–415) were retrieved with much higher frequency than the edges that were only found the subsampled networks of each group (Control subsample-6: 5–7, ME/CFS subsample-7: 6–9) (Figures S5B and S5C). To validate the “Unique” edges for each group, we use Receiver Operating Characteristic (ROC) curve analysis, comparing the frequency of subsampled edge retrieval for all “Unique” edges to the frequency of all “edges not found in the Networks” (the false-positive frequency of edge detection) (Figures S5D and S5E). Frequency cut-points were determined for each group based on the frequency at which sensitivity and specificity are approximately equal. Any “Unique” edges that were retrieved at a frequency below the cut-points were discarded. Five of the 96 candidate “Unique” Control edges and six of the 68 candidate “Unique” ME/CFS edges were below the frequency cut-point and were discarded.

For final validation, we cross-compared the retrieval frequency of the remaining “Unique” Control edges in the Control subsampled networks with the retrieval frequency of those edges in the ME/CFS subsampled networks (Figure S5F) and the frequency of “Unique” ME/CFS edges in the ME/CFS subsampled network with those edges in the Control subsampled networks (Figure S5G). Any that have less than a 100% difference in edge retrieval are discarded, unless they show a sign change for a given edge, indicating associations exist in the opposite direction in each group (we note that in all subsampled ME/CFS networks and all subsampled Control networks that each time a given edge was retrieved, the sign of the partial correlation was 100% consistent within each group-always negative or always positive within that group). For the remaining 91 “Unique” Control edges, 48 of them were not retrieved by any of the 1000 ME/CFS subsampled networks (Frequency = 0), 76 were retrieved in ≤ 10/1000 ME/CFS networks, 89 were retrieved in ≤ 100/1000 ME/CFS networks, and all but three were well below the frequency cut-point for the ME/CFS subsample. However, these three edges showed a large frequency differential for edge retrieval between the groups (>100% difference) and were therefore retained. For the remaining 62 “Unique” ME/CFS edges, 44 were not retrieved in the Control subsampled networks (Frequency = 0), 58 were retrieved in < 10/1000 Control networks, 61 were retrieved in < 100/1000 Control Networks. One edge was found in > 100 Control Networks, but still below the frequency cut-point for Control edges. This edge (*Escherichia coli*-*Intestinimonas butyriciproducens*) had a sign differential; it was negatively correlated in all of the 348 ME/CFS subsampled networks in which it was retrieved and positively correlated in all the 118 Control subsampled networks. Such a sign change was extremely rare in this dataset and may be of biological relevance. One “Unique” ME/CFS edge showed less than a 100% difference in edge retrieval frequency and was also discarded (red asterisk in Figure S5G). Validated “Unique” edges for Controls (91 edges) consisted of 83 positive interactions and 8 negative interactions that formed a single fully connected network subcomponent with 80 species represented in the subnetwork (Figure S4D; Table S7). The “Unique” bacterial interactions found in healthy controls are not found in ME/CFS. Thus, dysbiosis in ME/CFS is reflected not only by the gain of “Unique” edges, but also by the loss of many interactions found in healthy controls. The validated “Unique” edges for ME/CFS (61 edges) consisted of 49 positive interactions and 12 negative interactions that formed 9 separate network subcomponents with 70 species represented. The two largest “Unique” ME/CFS subcomponents consisted of 25 nodes and 24 edges and 15 nodes and 14 edges, respectively (Figure S4E; Table S7).

Finally, we create two “Aggregated” Networks by combining the validated unique edges from the “Control Network” and the validated unique edges from the “ME/CFS Network” with the “Common Network” edges to obtain the final “Aggregated Control” (AggCN) (Figure 5A) and “Aggregated ME/CFS” (AggMN) (Figure 5B) networks for comparison. A schematic of our strategy is shown in Figure 4E. All networks were constructed in Cytoscape v3.9.1. Global network properties and node centrality metrics were determined with the built in NetworkAnalyzer^101^ and CytoHubba^102^ apps in Cytoscape. Neighbor shift (NESH) scores were obtained from NetShift.^44^ Modules in the aggregated networks were identified from the unweighted networks using a fast greedy algorithm implemented in Cytoscape with clusterMaker2.^103^ Differences in local (node) centrality measures were assessed based on the degree difference (Δ: AggMN node centrality – AggCN node centrality). The Jaccard Index was calculated between network edges (edge Jaccard index) to measure the similarity between networks. The node Jaccard index was used to measure the similarity between community modules. Zi-Pi analyses on the aggregated networks were implemented with a published script in R.^104^ Zi-Pi plots categorize species as “Network hubs” (Zi>2.5, Pi>0.62), connecting to many species both within and between modules; “Module hubs” (Zi>2.5, Pi≤0.62), connecting to many species in their own module but with low inter-module connectivity; “Connectors” (Zi≤2.5, Pi>0.62), connecting to many species in other modules but with low intra-module connectivity; and “Peripherals” (Zi≤2.5, Pi≤0.62), connecting to few species and mostly within their own module.^104^ Differences in the frequency of positive and negative edges in the second order network neighborhood of *F. prausnitzii* were assessed with Fisher’s Exact Test.

#### Microbiome Multivariable Association with Linear Models (MaAsLin2)

Microbiome Multivariable Association with Linear Models (MaAsLin2 1.0.0 R package), which employs generalized linear models, was used to identify differentially abundant bacterial species between groups.^38^ Four models were assessed: Model 1, comparing ME/CFS vs. healthy controls; Model 2, ME/CFS without sr-IBS vs. healthy controls without sr-IBS; Model 3, ME/CFS with sr-IBS vs. healthy controls without sr-IBS; and Model 4, ME/CFS with sr-IBS vs. ME/CFS without sr-IBS. All models were adjusted for covariates [sr-IBS diagnosis (Model 1 only), site of recruitment, sex, BMI, race and ethnicity, age, antibiotic use within 6–12 weeks of testing, probiotic supplement use, and prebiotic supplement use]. Continuous measures (BMI and sex) were standardized to z-scores. Use of prescription narcotics, antidepressants and sample sequencing depth were tested in each model but did not affect the results and were therefore not included in the final models.

MaAsLin2 was also used to evaluate within-group associations between bacterial species abundance and z-score standardized SCFAs (individual molar proportions), as well as, between bacterial species and z-score standardized MFI scores (individual dimension scores and total scores), adjusting for covariates.

All analyses were carried out with the default linear model (LM) method, and the unrarefied species-level relative abundance table (after removal of structural zeros) was arcsine square root (AST) transformed and filtered by abundance and prevalence (minimum abundance of 0.01%; min prevalence of 20%). For between-group analyses, the false discovery rate (FDR) was controlled with the Benjamini-Hochberg procedure, and adjusted p-values (q-values) < 0.05 were considered statistically significant. For within-group analyses, the FDR was controlled at p < 0.01. The coefficient of each regression was used to interpret the direction and effect size of the association.

#### Machine learning classifiers

MaAsLin2 identified twelve bacterial species whose relative abundance differed between ME/CFS cases and healthy controls, with adjustment for covariates. Therefore, we investigated the potential of these twelve species for classifying ME/CFS. Five state-of-the-art machine learning classifiers, generalized linear model (GLM), Random Forest (RF), Gradient Boosting Method (GBM), Naive Bayes (NB), and Linear discriminant analysis (LDA), were evaluated and compared for classification performances. A staged approach was used to identify the best performing models and test their generalizability: 1. “exhaustive search” of all combinations of the twelve potential biomarkers within our primary dataset, 2. validation of performance across geographic sites of patient recruitment, and 3. test for generalizability in an external validation dataset.

As high dimensionality can lead to overfitting of machine learning models and optimal combinations of biomarkers can improve performance, we first employed the “exhaustive search” method (evaluating all possible combinations that can be iterated from the twelve bacterial species) to determine optimal biomarker combinations from these twelve bacterial species with each ML classifier in our primary dataset (n = 91 controls, n = 106 cases). For each iteration, our primary dataset was randomly split into a training set (80%) and a testing set (20%). In addition, the models were trained with 10-fold cross-validation. To accurately assess the performance and select the best models with biomarker combinations, the random resample process was repeated 20 times, and the median AUC score (Area Under the Curve) was obtained to represent the performance of classification. Combinations of bacterial species biomarkers were ranked by the AUC score for each classifier. The multicollinearity of selected bacterial species was also checked to avoid adverse effects on the estimated coefficients of the model.

Optimal classifiers should be accurate across geography. Since the subjects in our cohort were recruited from five different locations across the U.S, we also assessed the performance of ML classifiers at different geographic cohort sites to examine the robustness of the model. To assess performance independently by geographic site, individuals from each specific geographic site were used as the testing set, while the combined individuals from the four remaining geographic sites were used as the training set (note: Florida was excluded as a test set due to small sample size, but was included in each training set). Again the “exhaustive search” method was applied to identify high-performing combinations (high AUC) of bacterial species for the 4 geographic sites included as test sets. Top-ranked models obtained from random splitting (80% training, 20% testing) of the primary dataset and those obtained from geographic site splitting were compared to identify the best performing combination of bacterial species based on the score with each classifier.

To explore the generalizability of our classifiers, we tested the best performing classifiers using an external validation metagenomic dataset (Chronic Fatigue Initiative cohort, CFI),^24^ consisting of 50 healthy controls and 50 ME/CFS cases, sampled years prior (June 2014-October 2014) to our primary dataset. The assessment was conducted with 10-fold cross-validation with 20 randomizations as well, and the median AUC score from 20 randomized repeats was used to represent the performance of prediction.

#### Differential gut bacterial metabolic pathway modules

KEGG ontology profiles, determined using FMAP, were assigned to gut metabolic module profiles using GOmixer.^40^ Analyses focused on the top 2000 most abundant bacterial KEGG orthologs. Statistically over/under-represented gut metabolic modules between ME/CFS and controls were determined using GOmixer, which applies a Wilcoxon rank-sum test and the Benjamini-Hochberg false discovery rate (FDR) to correct for multiple testing. Data were scaled and the mean differences in pathways and modules were considered significant at an FDR adjusted p-value < 0.1.

#### Regression analyses

Fecal bacterial alpha diversity, qPCR quantities of fecal bacteria and the *but* gene, fecal SCFAs concentrations and metagenomic content of genes involved in butyrate synthesis pathways were all assessed with generalized linear regressions. All models were adjusted for covariates (sr-IBS diagnosis, testing site, sex, BMI, race and ethnicity, age, antibiotic use within 6–12 weeks of testing, probiotic supplement use and prebiotic supplement use). Use of prescription narcotics and antidepressants were tested in each model but did not affect the results and were therefore not included in the final models. Data distribution was evaluated using histograms for each outcome, and a variety of linear models were chosen after model fit was tested utilizing the BIC measure. For count data a negative binomial and Poisson regression were evaluated, for continuous data with a correct assumption of a normal distribution a linear regression was used, and for continuous data with a non-normal distribution a generalized linear regression with Gamma distribution with log link model was compared with a lognormal generalized linear regression model. The SCFA concentrations were assessed with a generalized linear regression with Gamma distribution with log link. The alpha diversity measures of Shannon index and Evenness were log transformed and were evaluated with a linear regression assuming normal distribution. The observed species measure was assessed using a negative binomial generalized linear model. The metagenomic content for each butyrate-producing gene in counts per million (CPM) was estimated with a generalized linear regression with Gamma distribution with log link. Finally, the RT-qPCR data in copies/gram feces was evaluated with a generalized linear regression with Gamma distribution with log link. For the generalized linear models with Gamma distribution with log link and the negative binomial generalized linear model, the exponent of the estimate is the fold change, and the coefficients of linear regressions assuming normal distribution indicates the linearity of the association between the predictors and outcome. For each regression an interaction term between ME/CFS and sr-IBS was tested to determine if the relationship between ME/CFS and the outcome was altered significantly by sr-IBS status. When the interaction term was not significant, it was removed from the final models. The interaction term was only significant for SCFAs models, and is shown in the result. Stratified analyses were conducted to evaluate the nature of the interaction in these models. Results were considered significant for predictor variables for all regression models when p-value < 0.05.

## Supplementary Material

Table S1

Table S2

Supplementary Figure Legends (all)

Table S3

Table S4

Table S5

Table S6

Table S7

## Figures and Tables

**Table 1.** Cohort characteristics

	ME/CFS (n = 106)	Control (n = 91)	p value
Age (mean ± SD)	47.8 ± 13.7	47.0 ±14.1	0.78^a^
BMI (mean ± SD)	26.1 ±5.2	25.2 ±4.7	0.31^a^
Sex (male|female)	31|75	22|69	0.52^b^
sr-IBS (yes|no)	35|71	3|88	<0.001^b^
Antibiotic use, 6–12 weeks (yes|no)	13|93	5|86	0.14^b^
Prebiotic supplement use (yes|no)	11|95	2|89	0.02^b^
Probiotic supplement use (yes|no)	41|65	10|81	<0.001^b^
Anti-depressant use (yes|no)	41|65	12|79	<0.001^b^
Narcotic-pain relievers (yes|no)	24|82	2|88^d^	<0.001^b^
Duration of ME/CFS >3 years (yes|no)	86|8^e^	-	-

Site			

Palo Alto, CA	19(18%)	18(19%)	0.30^c^
Salt Lake City, UT	32 (30%)	32 (35%)	
Incline Village, NV	23(22%)	17(19%)	
New York, NY	17(16%)	19(21%)	
Miami, FL	15(14%)	5(6%)	

Race			

Hispanic	6 (6%)	3 (3%)	0.40^c^
White and non-Hispanic	93 (88%)	85 (94%)	
Non-White and non-Hispanic	7 (6%)	3 (3%)	

MFI scores (mean ± SD)			

General fatigue	83.3 ±20.3	22.4 ±19.5	<0.001^a^
Physical fatigue	80.7 ±20.4	17.3 ±17.8	<0.001^a^
Mental fatigue	60.3 ±23.6	19.6 ±21.2	<0.001^a^
Reduced activity	74.9 ±22.7	16.7 ±19.8	<0.001^a^
Reduced motivation	48.4 ±26.3	20.7 ±23.5	<0.001^a^

a p values based on Mann-Whitney U test.

b p values based on Fisher’s exact test.

c p values based on chi-squared test.

d One missing response.

e Twelve missing response.

**Table 2.** Generalized linear regression showing the relationship between ME/CFS status and IBS status with alpha diversity metrics, fecal quantities of bacterial taxa, the *but* gene by qPCR, and metagenomic gene content in the acetyl-CoA pathway

		Alpha diversity metrics			qPCR results				
			
		Shannon	Evenness	Observed species	*Roseburia/Eubacterium*		*F. prausnitzii*	Total 16S/ACN	*but* gene
ME/CFS	β or fold change	−0.02	−0.02	0.98	0.56		0.61	2.08	0.51
	95% CI	−0.05–0.00	−0.04–0.00	0.93–1.03	0.33–0.93		0.38–0.98	1.77–2.84	0.28–0.93
	p value	0.103	0.096	0.481	0.026^a^		0.043^a^	<0.001^a^	0.028^a^
sr-IBS	β or fold change	−0.03	−0.03	1.01	0. 69		0.74	0.75	0.63
	95% CI	−0.06–0.00	−0.06–0.00	0.94–1.07	0.38–1.28		0.42–1.31	0.37–1.09	0.30–1.28
	p value	0.054	0.032^a^	0.865	0.245		0.301	0.132	0.203

		Metagenomics gene content in the acetyl-CoA pathway							
		
		*thl*	*bhbd*	*cro*	*bcd*	*etfA*	*etfB*	*but*	*buk*

ME/CFS	fold change	0.86	0.81	0.80	0.85	0.84	0.85	0.75	0.93
	95% CI	0.72–1.03	0.68–0.96	0.68–0.95	0.73–1.00	0.74–0.95	0.75–0.96 0.60–0.90	0.84–1.04
	p value	0.087	0.014^a^	0.008^a^	0.043^a^	0.005^a^	0.007^a^	0.003^a^	0.180
sr-IBS	fold change	1. 02	1.08	1.07	1.06	1.03	1.03	1.08	0.94
	95% CI	0.83–1.25	0.88–1.32	0.88–1.31	0.88–1.28	0.89–1.19	0.89–1.19 0.90–1.40	0.83–1.07
	p value	0.874	0.465	0.473	0.522	0.731	0.675	0.493	0.345

Results for each outcome variable are estimated using linear regression (Shannon and evenness) a negative binomial generalized linear model (observed species) or generalized linear regression with a Gamma distribution with log link (all other models). All models are adjusted for covariates.

Full regression tables are presented in Table S1.

The regression coefficient (β, for Shannon and Evenness) or fold change (all other models), 95% confidence intervals (CIs), and p values are shown for each model.

a Significant predictors (p < 0.05).

**Table:** KEY RESOURCES TABLE

REAGENT or RESOURCE	SOURCE	IDENTIFIER
Biological samples		

Human Fecal Samples	This paper	N/A

Chemicals, peptides, and recombinant proteins		

AL Buffer	QIAgen	cat. # 19075
pGEM T-Easy vector	Promega	cat. # PR-A1360
TaqMan Universal PCR Master Mix	Applied Biosystems	cat. # 4304437
SYBR Green PCR master mix	Applied biosystems	cat. #4309155
tert-butyl methyl ether	Sigma	cat# 306975

Critical commercial assays		

KAPA Hyper Prep kit	Kapa Biosystems	cat. # 07962363001(KK8504)
Gel Extraction kit	QIAgen	cat. # 28706X4
pGEM T-Easy vector	Promega	cat. # PR-A1360
Pure Link Plasmid Extraction Kit	Invitrogen	cat. #K210010
QIAmp DNA Stool Mini Kit	QIAgen	cat. #51504

Deposited data		

Raw and analyzed data	This paper	SRA: PRJNA751448

Oligonucleotides		

Primer total 16s quantification: TotalbacF 5’-TCCTACGGGAGGCAGCAGT-3’	Nadkarni et al.	N/A
Primer total 16s quantification: TotalbacR 5’-GGACTACCAGGGTATCTAATCCTGTT-3’	Nadkarni et al.	N/A
Probe total 16s quantification: TotalbacProbe 5’-FAM CGTATTACCGCGGCTGCTGG CA-BHQ-3’	Nadkarni et al.	N/A
Primer *but* gene quantification: BcoATF: 5’-GCIGAICATTTCACITGGAAYWSITGGCAY ATG-3’	Louis and Flint	N/A
Primer *but* gene quantification: BcoATR:5’-CCTGCCTTTGCAATRTCIACRAANGC-3’	Louis and Flint	N/A
Primer Roseburia/Eubacterium 16s quantification: RoseEub16sF1 5’-CGKACTAGAGTGTCGGAGG −3’	Ramirez-Farias et al.	N/A
Primer Roseburia/Eubacterium 16s quantification: RoseEub16sF2 5’-GTCATCTAGAGTGTCGGAGG-3’	Ramirez-Farias et al.	N/A
Primer Roseburia/Eubacterium quantification: RoseEub16sR 5’-AGTTTYATTCTTGCGAACG-3’	Ramirez-Farias et al.	N/A
Primer Faecalibacterium prausnitzii 16s quantification: FPR16sF 5’-GGAGGAAGAAGGTCTTCGG-3’	Ramirez-Farias et al.	N/A
Primer Faecalibacterium prausnitzii 16s quantification: FPF16sR 5’-AATTCCGCCTACCTCTGCACT-3’	Ramirez-Farias et al.	N/A

Software and algorithms		

GraphPad Prism 7.0	Graphpad	https://www.graphpad.com/
R Software	R Core Team	https://www.r-project.org/
Cutadapt	Matrin et al.^88^	https://cutadapt.readthedocs.io/en/stable/
FastQC	Babraham Bioinformatics	https://www.bioinformatics.babraham.ac.uk/projects/fastqc/
PrinSEQ	Schmieder et al.^89^	http://prinseq.sourceforge.net/
Bowtie2	Langmead and Salzberg^90^	http://bowtie-bio.sourceforge.net/bowtie2/index.shtml
Kraken2	Lu et al.^91^	https://ccb.jhu.edu/software/kraken2/
Bracken	Lu et al.^92^	https://ccb.jhu.edu/software/Bracken
ANCOM-II	Kaul et al.^93^	https://github.com/FrederickHuangLin/ANCOM-Code-Archive
QIIME2	Bolyen et al.^94^	https://qiime2.org/
FMAP	Kim et al.^95^	https://github.com/jiwoongbio/FMAP
NetShift	Kuntal et al.^44^	https://web.rniapps.net/netshift/
MaAsLin2	Mallick et al.^38^	https://huttenhower.sph.harvard.edu/maaslin/
GOmixer	Darzi et al.^40^	https://raeslab.org/software/omixer.html
AGS-and-ACN-tools	Pereira-Flores et al.^39^	https://github.com/pereiramemo/AGS-and-ACN-tools
FlashWeave	Tackmann et al.^43^	https://github.com/meringlab/FlashWeave.jl
Cytoscape v3.9.1	Cytoscape Team	https://cytoscape.org/

## Highlights

- ME/CFS patients have substantial gut microbiome dysbiosis
- Bacterial abundances, functions, SCFAs and species interactions deviate in ME/CFS
- Reduced *F. prausnitzii* and *E. rectale* in ME/CFS may contribute to butyrate deficiency
- Low *F. prausnitzii* abundance correlates with more severe fatigue symptoms in ME/CFS
