# Supplementary material for: Deficient butyrate-producing capacity in the gut microbiome is associated with bacterial network disturbances and fatigue symptoms in ME/CFS
Source: Cell Host Microbe. Author manuscript; Available in PMC 2023 May 15. (PMC10183837; doi:10.1016/j.chom.2023.01.004)
Supplement: Supplementary Figure Legends (all) [file NIHMS1884352-supplement-Supplementary_Figure_Legends__all_.pdf]

Cell Host & Microbe, Volume *31*

Supplemental information

Deficient butyrate-producing capacity in the gut microbiome is associated with bacterial network disturbances and fatigue symptoms in ME/CFS

Cheng Guo, Xiaoyu Che, Thomas Briese, Amit Ranjan, Orchid Allicock, Rachel A. Yates, Aaron Cheng, Dana March, Mady Hornig, Anthony L. Komaroff, Susan Levine, Lucinda Bateman, Suzanne D. Vernon, Nancy G. Klimas, Jose G. Montoya, Daniel L. Peterson, W. Ian Lipkin, and Brent L. Williams

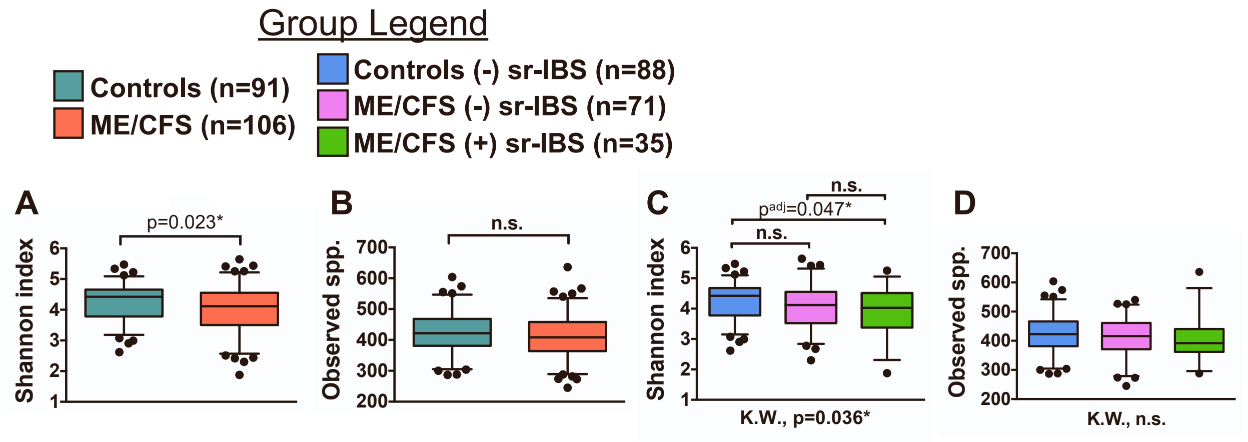

Figure S1. Gut microbiome alpha diversity, Related to Figure 1B-C. Box-and-whiskers plots showing the distribution of microbiome alpha diversity (Shannon diversity index and Observed species) between ME/CFS and healthy controls (**A, B**) and among stratified groups for healthy controls without (-) sr-IBS, ME/CFS subjects without (-) sr-IBS, and ME/CFS subjects with (+) sr-IBS (**C, D**). Box-and-whiskers plots represent the interquartile ranges (25th through 75th percentiles, boxes), medians (50th percentiles, bars within the boxes), the 5th and 95th percentiles (whiskers below and above the boxes), and outliers beyond the whiskers (closed circles). Statistical significance was determined based on two-tailed p-values from the Mann-Whitney U test (**A, B**). For stratified analyses, significance was first determined based on the Kruskal-Wallis test (K.W., results shown below each figure in **C, D**). If significant ($p < 0.05$) based on K.W., then between-group significance was determined based on the Mann-Whitney U test with multiple testing (Bonferroni) correction (p^{adj}-value). n.s. = not significant; * = p or $p^{adj} < 0.05$.

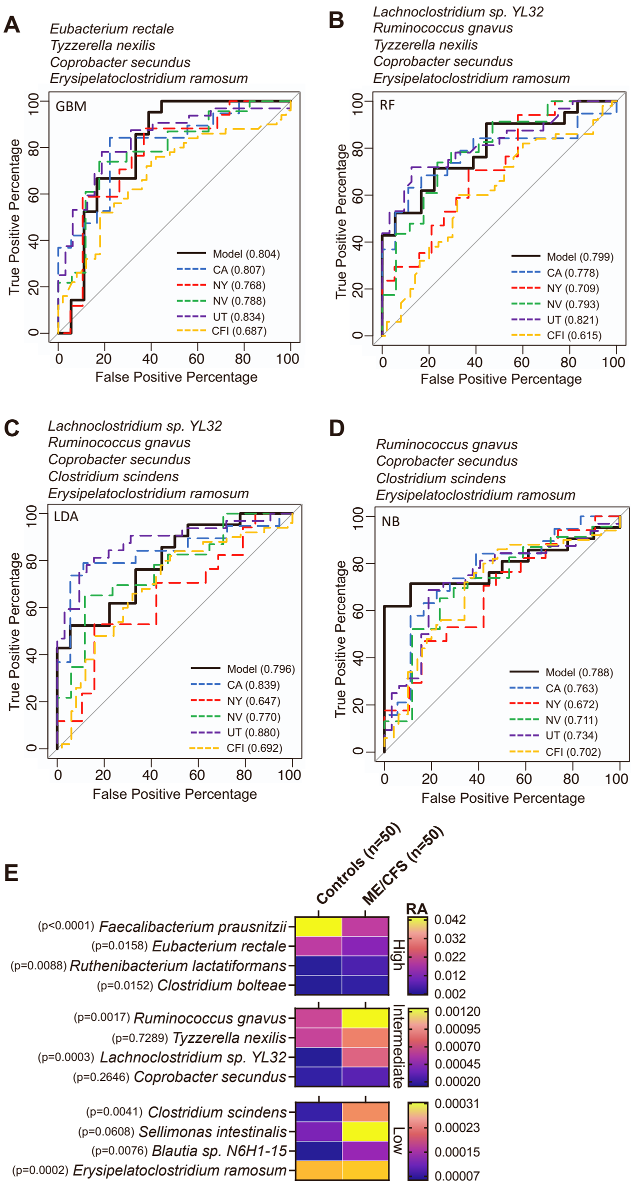

Figure S2. Additional results for the performance and assessment of machine learning classifiers, Related to Figure 2B-C. (**A-D**) Gradient Boosting Method (**A**: GBM), Random Forest (**B**: RF), Linear discriminant analysis (**C**: LDA), and Naive Bayes (**D**: NB) ROC curves for classification of ME/CFS based on the bacterial species indicated above each graph. AUC values are shown for the primary dataset in this study (Model), for within-study testing across geographic sites (CA, NY, NV, UT), and for generalization with an external validation dataset (CFI). (**E**) Heatmap showing the relative abundance (RA) of the twelve bacterial species in our external validation cohort (CFI) dataset, which were identified as differentially abundant between ME/CFS and controls after covariate adjustment and stratified analyses in our primary dataset. Species are divided into high, intermediate and low relative abundance. P-values based on the Mann-Whitney U test comparing each species between healthy controls and ME/CFS subjects are shown next to each species.

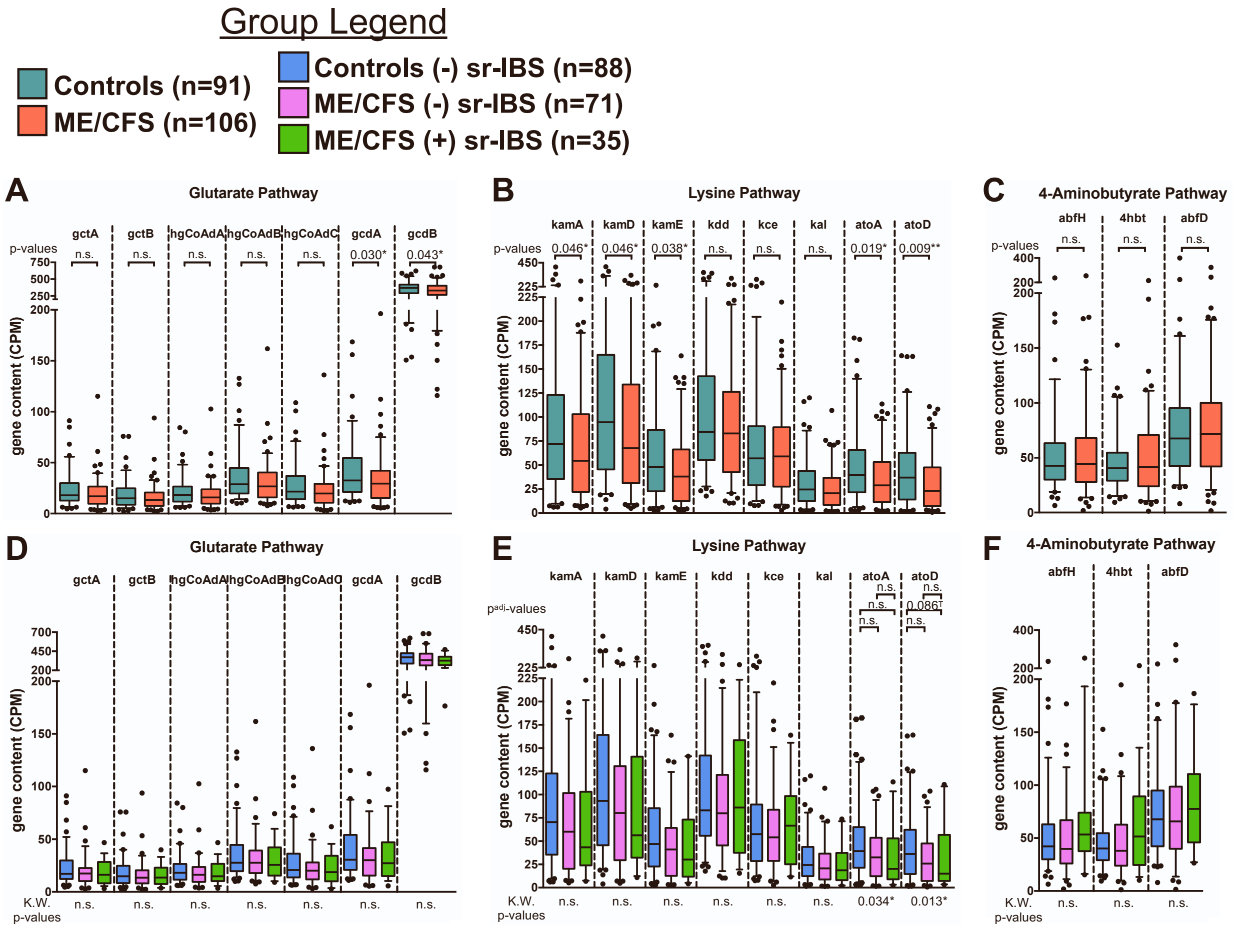

Figure S3. Metagenomic gene content of the fecal microbiota for genes in bacterial pathways of butyrate production, Related to Figure 3C-E. Box-and-whiskers plots showing the distribution of gene counts (CPM) for genes in the Glutarate (**A, D**), Lysine (**B, E**), and 4-Aminobutyrate (**C, F**) pathways of butyrate production between ME/CFS and healthy controls (**A-C**) and among stratified groups for healthy controls without (-) sr-IBS, ME/CFS subjects without (-) sr-IBS, and ME/CFS subjects with (+) sr-IBS (**D-F**). Box-and-whiskers plots represent the interquartile ranges (25th through 75th percentiles, boxes), medians (50th percentiles, bars within the boxes), the 5th and 95th percentiles (whiskers below and above the boxes), and outliers beyond the whiskers (closed circles). Statistical significance was determined based on two-tailed p-values from the Mann-Whitney U test for each gene (**A-C**). For stratified analyses, significance was first determined based on the Kruskal-Wallis test for each gene (K.W., results shown below each figure in **D-F**). If significant ($p < 0.05$) based on K.W., then between group significance was determined based on the Mann-Whitney U test with multiple testing (Bonferroni) correction (p^{adj}-value). n.s. = not significant; * = p or $p^{adj} < 0.05$; ** = p or $p^{adj} < 0.01$; T = trend (p or $p^{adj} < 0.1$).

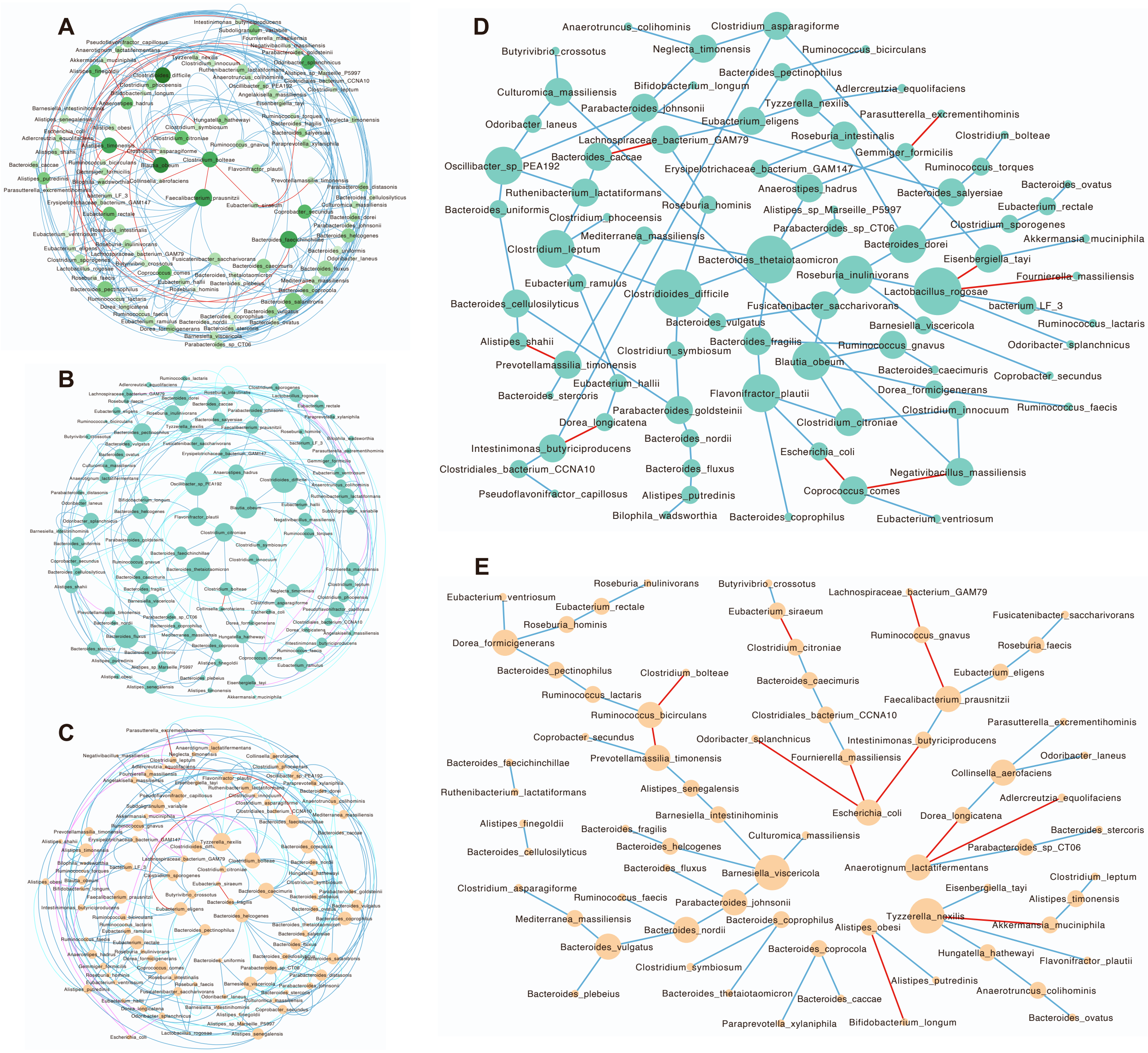

Figure S4. Intermediate Networks, Related to Figure 4E, Figure 5 and STAR Methods. (A-C) Bacterial species level co-abundance networks representing intermediate networks for the Common Network (**A**), the Control Network (**B**) and the ME/CFS Network (**C**). The networks are displayed in radial layout, with nodes sized by degree. In (**A**), node shade represents betweenness centrality (higher betweenness = darker nodes), and blue edges in the network denote positive correlations and red edges denote negative correlations. In (**B, C**), node color only represents the group from which the networks were built (**B**-Control and **C**-ME/CFS), and blue edges reflect positive and red edges negative correlations found in the “Common Network”, while aqua edges reflect positive and fuchsia negative edges that are “Unique” to that group. (**D, E**) Species co-abundance networks derived from the validated “Unique” Control edges (**D**, 91 edges) and the validated “Unique” ME/CFS edges (**E**, 61 edges). Nodes are sized by their degree centrality. Blue edges in the network denote positive correlations and red edges denote negative correlations.

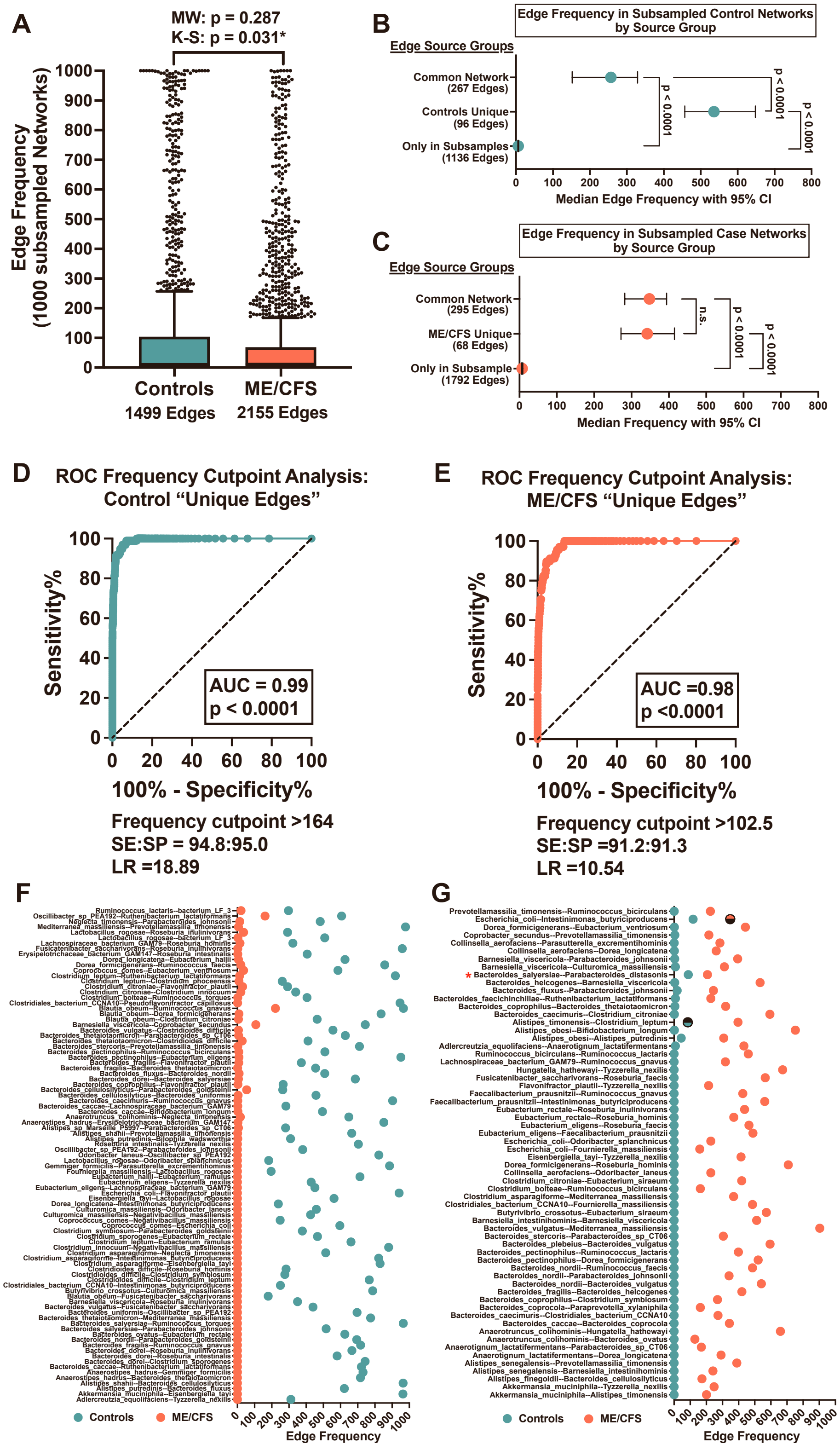

Figure S5. Evaluation of 1000 subsampled Flashweave networks for validation of unique edges found in the Control and ME/CFS co-abundance networks, Related

to Figure 4E, Figure 5 and **STAR Methods.** (**A**) Box-and-whiskers plots showing the distribution of edge frequency recovery of every edge from 1000 subsampled microbiome co-occurrence networks for healthy controls (n=75/subsample) and 1000 subsampled co-occurrence networks for ME/CFS cases (n=75/subsample). Box-and-whiskers plots represent the interquartile ranges (25th through 75th percentiles, boxes), medians (50th percentiles, bars within the boxes), the 5th and 95th percentiles (whiskers below and above the boxes), and outliers beyond the whiskers (closed circles). Differences between groups for overall edge frequency distributions were assessed with both the Mann-Whitney U test and the Kolmogorov-Smirnov test. (**B, C**) Symbol plots showing the median edge retrieval frequency (filled circles) and the 95% confidence interval (CI, error bars) from 1000 Control (**B**) and 1000 Case (**C**) subsampled networks for edges that are present in the Common network, Unique edges found in the Control (**B**) or ME/CFS (**C**) networks and subsampled edges that were not present in either the Common or group-specific networks. Differences between the edge retrieval frequency distributions were assessed by the Mann-Whitney U test with multiple testing (Bonferroni) correction (p^{adj}-values are shown). (**D, E**) Receiver-Operating Characteristic (ROC) plots assessing the sensitivity and specificity of Unique Control network edges (**D**) and Unique ME/CFS network edges (**E**) based on their frequency of retrieval from the subsampled networks compared to all false positive edge retrieval frequencies (defined by all edges only found in the subsampled networks). The area under the curve (AUC), the associated p-value, the cutpoint and its associated sensitivity (SE) and specificity (SP) and likelihood ratio are shown with each graph. Note, cutpoints defined for each group resulted in removal of 5 Unique Control edges and 6 Unique ME/CFS edges, leaving 91 Unique Control edges and 62 Unique ME/CFS edges. (**F**) Scatter dot plot showing the frequency of edge retrieval from the Control subsampled networks for the 91 Unique Control network edges above the ROC cutpoint and cross-compared with the frequency of retrieval of those edges in the ME/CFS subsampled networks. (**G**) Scatter dot plot showing the frequency of edge retrieval from the ME/CFS subsampled networks for the 62 Unique Case network edges above the ROC cutpoint and cross-compared with the frequency of retrieval of those edges in the Control subsampled networks. Half-filled symbols in (**G)** denote edges for which the edge weighted sign differed for the partial correlation, the group with the half-filled circle denotes the group with the negative edge weight. The asterisk in (**G**) denotes an edge where the frequency difference was minimal between the two subsampled datasets and no sign change was observed. Therefore, this edge was removed from the final validated list of Unique Case edges (Final validated Unique edges: Control = 91, ME/CFS = 61).

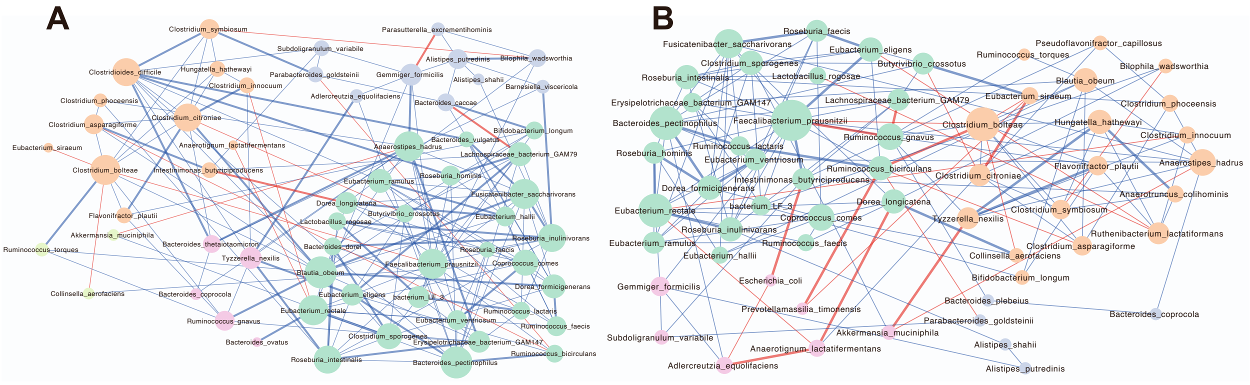

C

Total number of signed edges in the Aggregated Networks			
	Control	ME/CFS	Fisher's exact test P-value
Positive edges	359	325	0.4269
Negative edges	29	33	

Signed edges in the 2nd-order neighborhood of F. prausnitzii			
	Control	ME/CFS	Fisher's exact test P-value
Positive edges	164	131	0.0333 *
Negative edges	13	23	

Unique signed edges in the 2nd-order neighborhood of F. prausnitzii			
	Control	ME/CFS	Fisher's exact test P-value
Positive edges	41	16	0.0058 **
Negative edges	3	9	

Unique edges in the 2nd-order neighborhood of F. prausnitzii			
	Control	ME/CFS	Fisher's exact test P-value
Common edges	133	129	0.0585
Unique edges	44	25	

Figure S6. 2nd-order network of *F. prausnitzii*, Related to Figure 5F-G. Comparison of the 2nd-order network neighborhood surrounding *F. prausnitzii* in the Aggregated Control Network (**A**) and the Aggregated ME/CFS Network (**B**). Subgraph Networks were constructed from the 2nd-order nodes (Second neighbor species) from the vertex node for *F. prausnitzii* to evaluate how Unique edges from each group may influence network topology in the *F. prausnitzii* neighborhood. (**E**) Differences in the frequency of positive vs. negative and Common vs. Unique edges in the 2nd-order neighborhood were assessed with Fisher's exact test, the contingency table and p-values are shown for each test. * = $p < 0.05$, ** = $p < 0.01$

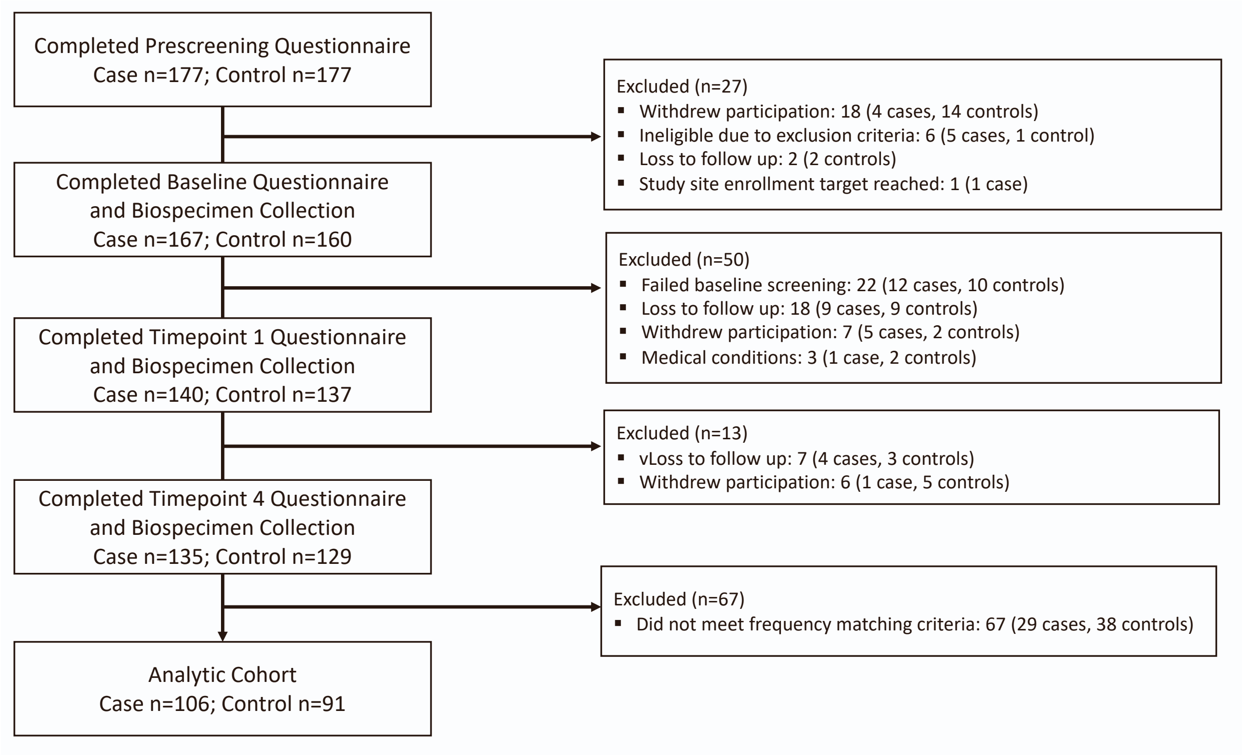

Figure S7. Flowchart for cohort sample selection, Related to STAR Methods.
